# Clinical practice guideline for renal hypouricemia (1st edition)

**DOI:** 10.1007/s13577-019-00239-3

**Published:** 2019-02-19

**Authors:** Akiyoshi Nakayama, Hirotaka Matsuo, Akira Ohtahara, Kazuhide Ogino, Masayuki Hakoda, Toshihiro Hamada, Makoto Hosoyamada, Satoshi Yamaguchi, Ichiro Hisatome, Kimiyoshi Ichida, Nariyoshi Shinomiya

**Affiliations:** 1 Department of Integrative Physiology and Bio-Nano Medicine, National Defense Medical College, 3-2 Namiki, Tokorozawa, Saitama 359-8513 Japan; 2 Medical Squadron, Air Base Group, Western Aircraft Control and Warning Wing, Japan Air Self-Defense Force, Kasuga, Japan; 3 Division of Cardiology, Sanin Rosai Hospital, Yonago, Japan; 4 Department of Cardiology, Japanese Red Cross Tottori Hospital, Tottori, Japan; 5 Department of Nutritional Sciences, Faculty of Human Ecology, Yasuda Women’s University, Hiroshima, Japan; 6 Department of Regional Medicine, Tottori University Faculty of Medicine, Tottori, Japan; 7 Department of Human Physiology and Pathology, Faculty Pharma-Science, Teikyo University, Tokyo, Japan; 8 Department of Urology, The Urinary Stone Medical Center, Kitasaito Hospital, Asahikawa, Japan; 9 Department of Genetic Medicine and Regenerative Therapeutics, Institute of Regenerative Medicine and Biofunction, Tottori University Graduate School of Medical Science, Tottori, Japan; 10 Department of Pathophysiology, Tokyo University of Pharmacy and Life Sciences, Tokyo, Japan

**Keywords:** Renal hypouricemia (RHUC), Clinical practice guideline (CPG), Evidence-based medicine (EBM), Exercise-induced acute kidney injury (EIAKI), Acute renal failure with severe loin pain and patchy renal ischemia after anaerobic exercise (ALPE)

## Abstract

Renal hypouricemia (RHUC) is a disease caused by dysfunction of renal urate reabsorption transporters; however, diagnostic guidance and guidelines for RHUC have been lacking, partly due to the low evidence level of studies on RHUC. This review describes a world-first clinical practice guideline (CPG) and its first version in English for this condition. It was developed following the “MINDS Manual for Guideline Development” methodology, which prioritizes evidence-based medicine. It was published in Japanese in 2017 and later translated into English. The primary goal of this CPG is to clarify the criteria for diagnosing RHUC; another aim is to work towards a consensus on clinical decision-making. One of the CPG’s unique points is that it contains textbook descriptions at the expert consensus level, in addition to two clinical questions and recommendations derived from a systematic review of the literature. The guidance shown in this CPG makes it easy to diagnose RHUC from simple blood and urine tests. This CPG contains almost all of the clinical foci of RHUC: epidemiology, pathophysiology, diagnostic guidance, clinical examinations, differential diagnosis, and complications, including exercise-induced acute kidney injury and urolithiasis. A CPG summary as well as a clinical algorithm to assist healthcare providers with a quick reference and notes from an athlete for both physicians and patients are included. We hope that this CPG will help healthcare providers and patients to make clinical decisions, and that it will promote further research on RHUC.

**Electronic supplementary material:**

The online version of this article (10.1007/s13577-019-00239-3) contains supplementary material, which is available to authorized users.

## Introduction/background

Renal hypouricemia (RHUC) is a pathological condition that is caused by faulty urate reabsorption transporters in the renal proximal tubular cells. It does not derive from congenital purine metabolism abnormalities or secondary hypouricemia. In this review, we display an overview and summary of our world-first clinical practice guideline (CPG) for RHUC (See Supplementary Material for the complete guideline).

Ever since Akaoka et al. [1] first reported a case study of RHUC in Japan in 1975, RHUC has been known to be relatively common in the Japanese population (approximately 0.3%) [2, 3]. RHUC is also reported in Jewish [4] and Roma [5] populations. Recent genetic studies have identified faults in the *URAT1*/*SLC22A12* [6] and *GLUT9*/*SLC2A9* [3] urate transporter genes as causes of RHUC type 1 and type 2 [7], respectively, but there are some patients who have no dysfunctional mutations in these genes, suggesting that there may be unknown causative genes. Nevertheless, healthcare providers lack awareness of RHUC, partly because there is no diagnostic guidance by expert consensus and no guidelines. This situation prompted us to develop the first ever CPG for RHUC. The primary goal of this CPG is, therefore, to clarify the criteria for diagnosing RHUC. Another goal is to work towards forming a consensus on clinical decision-making by enlightening healthcare providers.

## Methods used for developing the CPG

As our methodology, we adopted the Medical Information Network Distribution Service (MINDS) Manual for Guideline Development [8–10] which has been developed by the Japan Council for Quality Health Care to secure the broadest possible scientific basis. For example, three teams developed the guideline: the Guideline Supervising Committee, Guideline Developing Group (GDG), and Systematic Review Team (SRT), in addition to the Guideline Secretariat, chosen to prevent conflict of interest, as shown in the CPG. In the current edition, two clinical questions (CQs) are posed, based on key clinical issues: the diagnosis of RHUC and prevention of exercise-induced acute kidney injury (EIAKI, also known as acute renal failure with severe loin pain and patchy renal ischemia after anaerobic exercise: ALPE), which is one complication of RHUC. Before the publication of this CPG, a draft was evaluated by an external reviewer and public comments were invited. The Japanese version was published in 2017. This CPG has been translated into English and is here provided as Supplementary Material.

This CPG project was supported by grants from Japan’s Ministry of Health, Labour and Welfare (fiscal year 2014–2016).

## Textbook descriptions

There was no research on RHUC with sufficiently good evidence for CPG, such as randomized controlled trials (RCTs), but excluding all research reports on RHUC without exception due to low evidence levels would send the wrong message to clinical practitioners, and would also depart from the key aim of this guideline, which is to enlighten healthcare providers about RHUC. Therefore, “consensus levels” are added as expert opinions on each statement in the textbook description. This CPG contains almost all the clinical points relevant to RHUC: it covers epidemiology, pathophysiology, diagnostic guidance, clinical examinations, differential diagnosis, and complications. The summary of the textbook description is as follows.

**Fig. 1:**
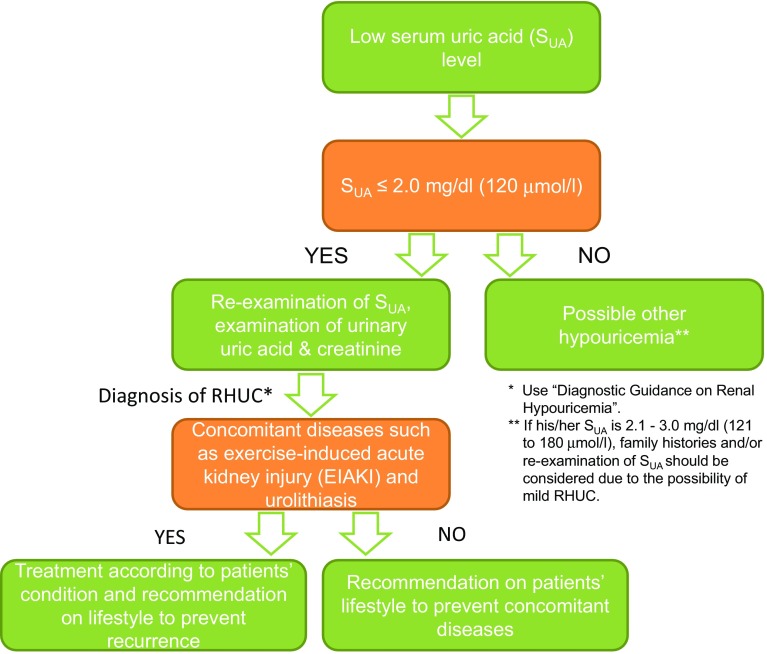
Clinical algorithm for renal hypouricemia (RHUC). If physicians detect a low serum uric acid (SUA) level, they should examine whether the patient’s SUA is lower than 2 mg/dl (120 µmol/l) or not. If it is, the physicians should reexamine the patient’s SUA level, because it sometimes varies according to their condition, and also test their urinary excretion of uric acid. Using these clinical data, diagnosis of RHUC is made along with the diagnostic guidance shown in Table 1. Physicians should treat patients appropriately if they suffer complications of RHUC, such as exercise-induced acute kidney injury (EIAKI) or urinary stones. If they do not have such complications, physicians should advise them that they are at risk of these complications and should therefore take action to prevent them. Because some mild RHUC patients show an SUA of 2.1–3.0 mg/dl, physicians should retest their SUA level and asking if there is a familial history of RHUC

The prevalence of RHUC is estimated to be approximately 0.2% in males and 0.4% in females in Japan [2, 3]. Although RHUC itself is asymptomatic, one of its characteristics is a low serum uric acid (SUA) level combined with increased renal excretion of uric acid. Figure 1 and Table 1, respectively, display the clinical algorithm and diagnostic guidance for RHUC. One notable characteristics of the diagnostic guidance is its simplicity: it enables a diagnosis of RHUC from simple blood and urine tests alone, and does not always require genetic examinations. Table 2 shows the differential diagnosis of hypouricemia. Of these, excluding drug-induced hypouricemia, the asymptomatic diseases that result in hypouricemia are only RHUC and xanthinuria. Xanthinuria is a rare disease with only a few 100 patients ever reported, and is distinguishable from other types of hypouricemia because it shows little urinary urate excretion [11].

**Table 1:** Diagnostic guidance for RHUC

Required factors: Confirming continuous findings of #1 and #2, while satisfying #3
#1 Hypouricemia with serum uric acid (*S*_UA_) level of ≤ 2.0 mg/dl (120 µmol/l)^a^
#2 Increased fractional excretion of uric acid (FE_UA_) and/or uric acid clearance (*C*_UA_)^b^
#3 Exclusion of other diseases that present hypouricemia as a symptom (Table 2)
Reference factors
(1) Mutations in the causative genes of RHUC (*URAT1/SLC22A12* and *GLUT9/ SLC2A9* genes)
(2) Past history of exercise-induced acute kidney injury (EIAKI)^c^
(3) Familial history of RHUC

^a^There is a possibility of mild RHUC even with an *S*_UA_ of 2.1–3.0 mg/dl (121–180 µmol/l). Repeated tests for Required factors #1 and #2 above are therefore desirable, especially when confirming any of the Reference factors (1) to (3) below

^b^The normal range of FE_UA_ and *C*_UA_ is 8.3 (5.5–11.1) % and 11.0 (7.3–14.7) ml/min, respectively

^c^Because *S*_UA_ is not always lower during onset of EIAKI, *S*_UA_ should be checked before onset (if possible) or after amelioration

**Table 2:** Differential diagnosis of RHUC (Diseases that cause hypouricemia)

1. Overexcretion-type hypouricemia
(1) Renal hypouricemia (RHUC) (2) Fanconi syndrome (3) Wilson’s disease (4) Syndrome of inappropriate secretion of antidiuretic hormone (SIADH) (5) Malignant tumor (6) Diabetes mellitus (7) Drugs (such as benzbromarone or probenecid) (8) Pregnancy (9) Intractable diarrhea
2. Underproduction-type hypouricemia
(1) Xanthinuria (type I, type II) (2) Molybdenum cofactor deficiency (3) Purine nucleoside phosphorylase deficiency (PNP deficiency) (4) PRPP synthetase hypoactivity (5) Idiopathic urate underproduction-type hypouricemia (6) Severe hepatic injury (7) Drugs (such as allopurinol) (8) Emaciation (malnutrition)

Most RHUC cases have been discovered by chance in health examinations that detect low SUA levels. Clinical signs of urolithiasis and EIAKI often lead to an initial diagnosis of RHUC. EIAKI cases sometimes need hemodialysis treatment, but generally show transient and recurrent acute kidney injury (AKI), and receive the standard treatment for AKI. Allopurinol, a xanthine oxidoreductase (XOR) inhibiter, was administered in some case reports [12–14] for preventive purposes, based on a hypothetic mechanism of its pathogenesis, although convincing evidence for its efficacy is lacking. For urinary stones, urinary alkalization using citrate compounds is an effective therapy [15], and drinking plenty of fluids is recommended for prevention.

## CQs and recommendations

Based on the experts’ opinions of the textbook description above, two key clinical issues were raised on the diagnosis of RHUC and prevention of EIAKI using the PICO (Patient/Population/Problem, Intervention, Comparison/Control/Comparator, and Outcome) framework. The first CQ (CQ1) is “Should individuals with a serum uric acid level of ≤ 2.0 mg/dl be considered for differential diagnosis of hypouricemia?”, and the second CQ (CQ2) is “Should XOR inhibitors be administered to prevent EIAKI in patients with RHUC?” Two independent SRT members in charge of each CQ conducted a literature search and two screenings. The survey uncovered 545 papers, of which 455 were adopted after screening for CQ1; and 43 of 54 papers passed CQ2. Based on the submitted systematic review report by SRT, GDG decided on the recommendations and their strength as shown in Table 3.

**Table 3:** List of Clinical Questions (CQs) and Recommendations

CQ1: Should individuals with a serum uric acid level of ≤ 2.0 mg/dl be considered for differential diagnosis of hypouricemia?
Recommendation 1: It is strongly recommended that such individuals be considered for differential diagnosis of hypouricemia.
CQ2: Should xanthine oxidoreductase (XOR) inhibitors be administered to prevent exercise-induced acute kidney injury (EIAKI) in patients with renal hypouricemia?
Recommendation 2: It is not yet possible to make a hard-and-fast rule. However, XOR inhibitors might prevent the onset or relapse of EIAKI. Administration of XOR inhibitors should therefore be decided in the light of its potential benefits and harms, especially for athletes and high-risk patients with a past history of EIAKI attacks.

## Applicability, patient’s views and future hopes

For applicability of the CPG, that is, for healthcare providers’ easy reference, a Brief Summary of the CPG, including Fig. 1 and Tables 1, 2 and 3, were added to the first page. Moreover, “Notes from an athlete” for the enlightenment of both physicians and patients is provided by a patient with RHUC, a female mixed martial arts fighter struggling with EIAKI.

As described above, the main purpose of publishing the CPG is to make this guideline available to healthcare providers, so that the actual prevalence of renal hypouricemia can be clarified and research on RHUC further promoted. It is hoped that this CPG will encourage the development of novel therapies as well as preventive methods for RHUC patients worldwide.

## Electronic supplementary material

Below is the link to the electronic supplementary material.

Supplementary material 1 (PDF 6184 KB)
