# Supplementary material for: Clinical practice guideline for renal hypouricemia (1st edition)
Source: Hum Cell. 2019 Feb 19;32(2):83–7. doi: 10.1007/s13577-019-00239-3 (PMC6437292; doi:10.1007/s13577-019-00239-3)

Clinical Practice Guideline for Renal Hypouricemia

[This is the official English version, translated from the original 1st Japanese edition]

Jointly Edited by:

- Renal Hypouricemia Research Team
- Japanese Society of Gout and Nucleic Acid Metabolism

2017 or
1 st
Edition

Brief Summary of This Guideline

Clinical Algorithm for Renal Hypouricemia (RHUC)

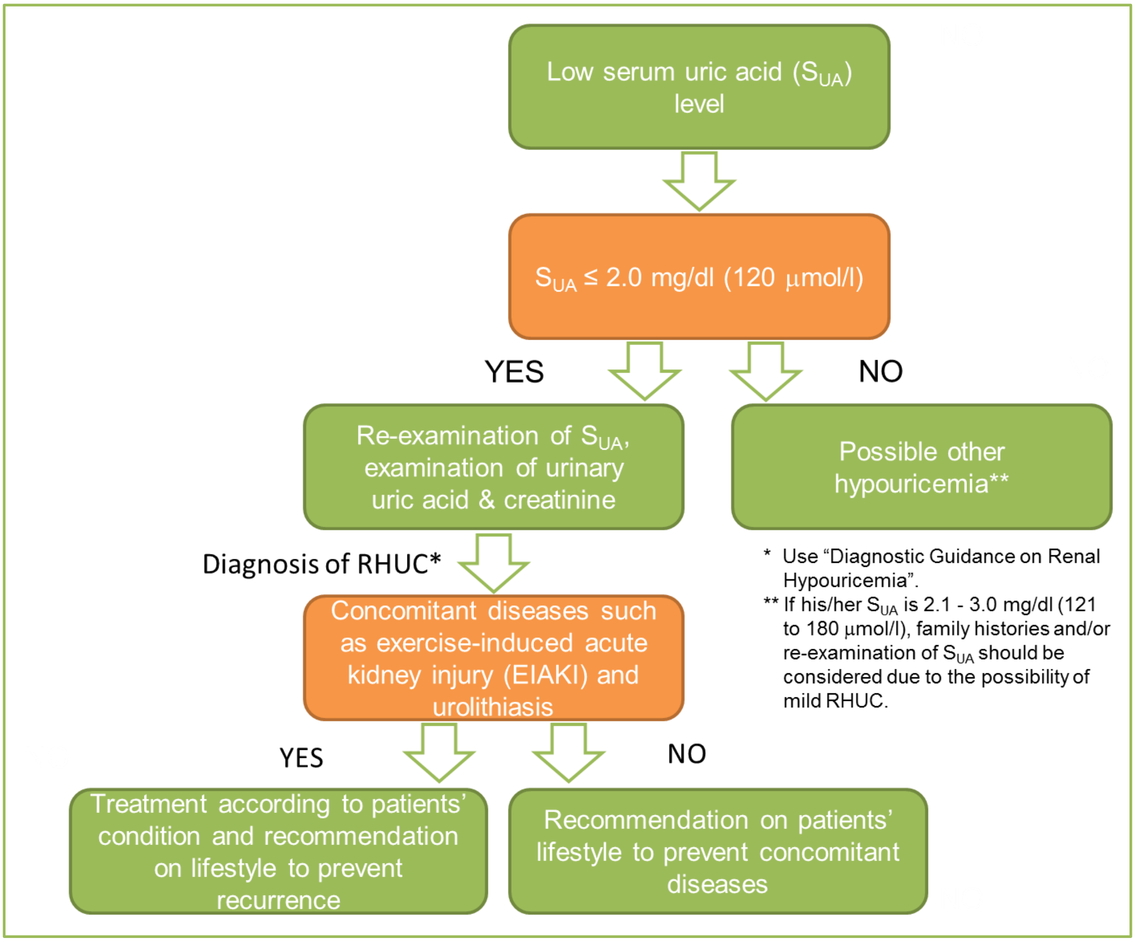

List of Clinical Questions (CQs) and Recommendations

**CQ1**	Should individuals with a serum uric acid level of ≤ 2.0 mg/dl be considered for differential diagnosis of hypouricemia?
**Recommendation 1**	We strongly recommend that such individuals be considered for differential diagnosis of hypouricemia.
**CQ2**	Should xanthine oxidoreductase (XOR) inhibitors be administered to prevent exercise-induced acute kidney injury (EIAKI) in patients with renal hypouricemia?
**Recommendation 2**	It is not yet possible to make a hard-and-fast rule.<br>However, it is possible that XOR inhibitors might prevent the onset or relapse of EIAKI. Administration of XOR inhibitors should therefore be decided in the light of its potential benefits and harms, especially for athletes and high-risk patients with a past history of EIAKI attacks.

Diagnostic Guidance on Renal Hypouricemia (RHUC)

Required factors

Confirming continuous findings of #1 and #2, while satisfying #3.	
#1	Hypouricemia with serum uric acid (S_{UA}) level of ≤ 2.0 mg/dl (120 μmol/l)*.
#2	Increased fractional excretion of uric acid (FE_{UA}) and/or uric acid clearance (C_{UA})**.
#3	Exclusion of other diseases that present hypouricemia as a symptom (Attached Table).

* There is a possibility of mild RHUC even with an S_{UA} of 2.1 - 3.0 mg/dl (121 - 180 μmol/l). Repeated tests for Required Factors #1 and #2 above are therefore desirable, especially when confirming any of the Reference Factors 1) to 3) below.

** The normal range of FE_{UA} and C_{UA} is 8.3 (5.5 - 11.1) % and 11.0 (7.3 - 14.7) ml/min, respectively.

Reference factors

1)	Mutations in the causative genes of RHUC (URAT1/SLC22A12 and GLUT9/ SLC2A9 genes).
2)	Past history of exercise-induced acute kidney injury (EIAKI)***.
3)	Familial history of RHUC.

*** Because S_{UA} is not always lower during onset of EIAKI, S_{UA} should be checked before onset (if possible) or after amelioration.

Attached Table: Differential Diagnosis of RHUC
(Diseases which cause hypouricemia)

1 Overexcretion-type hypouricemia

(1) Renal hypouricemia (RHUC)	(6) Diabetes mellitus
(2) Fanconi syndrome	(7) Drugs (such as benzbromarone and probenecid)
(3) Wilson's disease	(8) Pregnancy
(4) Syndrome of inappropriate secretion of antidiuretic hormone (SIADH)	(9) Intractable diarrhea
(5) Malignant tumor	

2 Underproduction-type hypouricemia

(1) Xanthinuria (type I, type II)	(5) Idiopathic urate underproduction-type hypouricemia
(2) Molybdenum cofactor deficiency	(6) Severe hepatic injury
(3) Purine nucleoside phosphorylase deficiency (PNP deficiency)	(7) Drugs (such as allopurinol)
(4) PRPP synthetase hypoactivity	(8) Emaciation (malnutrition)

Contents

Brief Summary of This Guideline………………2
Preface…………………………………………….………..6
Abbreviations and Convergent Terms…………8

Introduction

The Need for this Guideline and its Goals……………………….………10
Methods Used to Develop this Guideline..……………………………..…13

Chapter 1.

Clinical Algorithm for Renal Hypouricemia…………………….….……21

Chapter 2.

The Epidemiology of Renal Hypouricemia……………………….………22

Chapter 3.

The Pathophysiology of Renal Hypouricemia..…………………..……26

Chapter 4.

Guidance on the Diagnosis, Examination,
and Differential Diagnosis of Renal Hypouricemia……………31

Chapter 5.

Concomitant Disease of Renal Hypouricemia
(Exercise-Induced Acute Kidney Injury)……………………..……35

Chapter 6.

Concomitant Disease of Renal Hypouricemia (Urolithiasis)……….……39

Chapter 7.

Clinical Questions and Recommendations……………………….………44

Appendix

Notes from an Athlete………………………………………………….……50

Guideline Development Committee Members

Lead Researcher

Name	Affiliation	Specialty
**Nariyoshi SHINOMIYA**	Professor, Department of Integrative Physiology and Bio-Nano Medicine, National Defense Medical College, Japan.	Molecular Biology<br>Clinical Genetics

Members

(in alphabetical order. "*"indicates commissioned members of the Japanese Society of Gout and Nucleic Acid Metabolism)

Name	Affiliation	Specialty
**Masayuki HAKODA***	Professor, Department of Nutritional Sciences, Faculty of Human Ecology, Yasuda Women's University, Japan.	Epidemiology
**Toshihiro HAMADA**	Assistant Professor, Department of Regional Medicine, Tottori University Faculty of Medicine, Japan.	Geriatric Gerontology,<br>Clinical Hypertension,<br>Cardiovascular Medicine
**Ichiro HISATOME***	Professor, Department of Genetic Medicine and Regenerative Therapeutics, Institute of Regenerative Medicine and Biofunction, Tottori University Graduate School of Medical Science, Japan.	Biochemistry,<br>Cardiovascular Medicine,<br>Regenerative Medicine
**Makoto HOSOYAMADA***	Professor, Department of Human Physiology and Pathology, Faculty Pharma-Science, Teikyo University, Japan.	Pharmacology
**Kimiyoshi ICHIDA***	Professor, Department of Pathophysiology, Tokyo University of Pharmacy and Life Sciences, Japan.	Nephrology
**Hirotaka MATSUO**	Lecturer, Department of Integrative Physiology and Bio-Nano Medicine, National Defense Medical College, Japan.	Clinical Genetics,<br>Molecular Genetic Epi-demiology
**Akiyoshi NAKAYAMA**	Graduate Student, Department of Integrative Physiology and Bio-Nano Medicine, National Defense Medical College, Japan.	Molecular Biology
**Kazuhide OGINO**	Assistant Professor, Department of Clinical Laboratory, Tottori University Hospital, Japan.	Cardiovascular Medicine
**Akira OHTAHARA**	Chief Doctor, the Division of Cardiology, Sanin Rosai Hospital, Japan.	Clinical Hypertension
**Satoshi YAMAGUCHI***	Director, Department of Urology and the Urinary Stone Medical Center, Kitasaito Hospital, Japan.	Urolithiasis,<br>Urate Metabolism

External Reviewer

Name	Affiliation	Specialty
**Shori TAKAHASHI**	Former Secretary General, Japanese Society for Pediatric Nephrology;<br>Professor, Department of Pediatrics and Child Health, Nihon University School of Medicine, Japan.	Pediatric Nephrology

Preface Reasons for the Development of This Guideline

Renal hypouricemia is a cardinal pathological condition that is caused by faulty urate reabsorption by the kidney. Analyses of its causes have revealed two causative genes: urate transporter 1 (*URAT1/SLC22A12*) and glucose transporter 9 (*GLUT9/SLC2A9*). Renal hypouricemia caused by these genetic variants is respectively classified into renal hypouricemia type 1 and type 2, respectively. A small number of renal hypouricemia cases, however, have unidentified causes.

A research group on renal hypouricemia carried out a research project entitled "Studies to Investigate the Actual Prevalence Renal Hypouricemia Nationwide" (Lead researcher: Nariyoshi Shinomiya) from fiscal year 2010 to 2011, with a grant for Research on Measures for Intractable Diseases, funded by a Health and Labour Sciences Research Grant. It was continued, in fiscal years 2012 and 2013, as a single multicenter research group, forming part of "Research on Rare/intractable Diseases of the Kidney and Urinary Systems" (Lead researcher: Kazumoto Iijima) with a grant for Research on Measures for Intractable Diseases, funded by a Health and Labour Sciences Research Grant.

While progressively untangling the causes and pathogenesis of renal hypouricemia through these studies, we also noted that this disease is little understood nationwide, even by healthcare personnel. We therefore concluded that we should publicize renal hypouricemia more widely under a clear unified guideline that would help draw attention to the disease and foster the establishment of its treatment and prevention. This prompted us to develop a clinical practice guideline for renal hypouricemia as a multicenter research group under "Research to Develop Diagnostic Criteria/clinical Practice Guidelines on Rare/intractable Diseases of the Kidney and Urinary Systems (Lead researcher: Kazumoto Iijima)" with a grant for Research on Measures for Intractable Diseases, funded by a Health and Labour Sciences Research Grant. This was planned to be implemented in fiscal years 2014 to 2016.

As described above, the chief aims of developing this Guideline are to more widely publicize and draw attention to renal hypouricemia in the hope of encouraging better treatments and prevention measures for patients. There is, however, only a limited body of literature covering analysis of its causes and pathologies, case reports, and its diagnoses and treatments, which we cannot regard as a sufficient clinical foundation. The development of this Guideline is an important first step towards assembling the latest findings from researchers and healthcare providers nationwide, which can then be fed into the next and subsequent Guidelines. This version, therefore, although it incorporates the best and most recent standards, is far from the

last word on their use or their application to all patients. This Guideline, therefore, does not automatically overrule the discretion of healthcare providers in actual clinical settings. It is, however, the case that the Guideline Development Committee Members have done their very best, based on MINDS (the Medical Information Network Distribution Service) of the Japan Council for Quality Health Care.

This clinical practice guideline on renal hypouricemia from Japan is a world first. It is my sincere wish that researchers and healthcare providers will take this opportunity to use it to further develop their studies and clinical practice.

For the development of this clinical practice guideline, I am grateful to the Ministry of Health, Labour and Welfare for its grant support, to Prof. Kazumoto Iijima (Department of Pediatrics, Kobe University Graduate School of Medicine) the Lead researcher, who supervised "Research on Rare/intractable Diseases of the Kidney and Urinary Systems" with a grant for Research on Measures for Intractable Diseases, funded by a Health and Labour Sciences Research Grant, and to Prof. Tatsuo Hosoya, the Chief Director of the Japanese Society of Gout and Nucleic Acid Metabolism (JSGNAM), as well as other directors and persons associated with JSGNAM, who generously accepted our proposal to co-edit this Guideline. Moreover, I would like to express my deepest gratitude to Prof. Shori Takahashi, the former Secretary General of the Japanese Society for Pediatric Nephrology, who provided valuable input as the external reviewer of this Guideline, and to all those who reviewed it during the public comments stage. I would also like to thank all the Guideline Development Committee Members, who expended so much time and effort on this project, and to Ms. Mei Yamaguchi, a renal hypouricemia patient who gave us extremely useful advice.

Nariyoshi Shinomiya

Representative of the Guideline Development Committee Members

February 2017

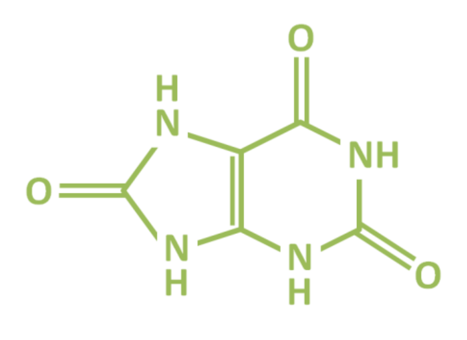

Abbreviations and Convergent Terms

Abbreviation	Meaning
**ADP**	adenosine diphosphate
**AKI**	acute kidney injury
**ALPE** → **Converged with "EIAKI"**	acute renal failure with severe loin pain and patchy renal ischemia after anaerobic exercise
**AMP**	adenosine monophosphate (adenylic acid)
**APRT**	adenine phosphoribosyltransferase
**ARF** → **Converged with "AKI"**	acute renal failure
**ATP**	adenosine triphosphate
**C_{Cre}**	creatinine clearance
**C_{UA}**	uric acid clearance
**C_{UA} / C_{Cre}** → **Converged with "FE_{UA}"**	uric acid clearance / creatinine clearance ratio
**CKD**	chronic kidney disease
**EIAKI**	exercise-induced acute kidney injury
**EIARF** → **Converged with "EIAKI"**	exercise-induced acute renal failure
**FE_{UA}**	fractional excretion of uric acid
**GLUT9/SLC2A9**	glucose transporter 9 / solute carrier family 2, member 9
**GDP**	guanosine diphosphate
**GMP**	guanosine monophosphate (guanylic acid)
**GTP**	guanosine triphosphate
**HPRT**	hypoxanthine phosphoribosyltransferase

**IMP**	inosine monophosphate (inosinic acid)
**MIM**	Mendelian Inheritance in Man database
**NSAIDs**	Non-steroidal anti-inflammatory drugs
**OMIM** → **Converged with "MIM"**	Online Mendelian Inheritance in Man database
**PNP**	purine nucleoside phosphorylase
**PRES**	posterior reversible encephalopathy syndrome
**PRPP**	phosphoribosyl pyrophosphate
**RHUC**	renal hypouricemia
**RHUC1**	renal hypouricemia type 1
**RHUC2**	renal hypouricemia type 2
**rs (numbers)**	reference SNP ID number
**SNP**	single nucleotide polymorphism
$\mathbf{S_{Cre}}$	serum creatinine level
$\mathbf{S_{UA}}$	serum uric acid level
**URAT1/SLC22A12**	urate transporter 1 / solute carrier family 22, member 12
$\mathbf{U_{Cre}}$	urinary creatinine level
$\mathbf{U_{UA}}$	urinary uric acid level
**UOX**	urate oxidase (uricase)
**XDH** → **Converged with "XOR"**	xanthine dehydrogenase
**XO** → **Converged with "XOR"**	xanthine oxidase
**XOR**	xanthine oxidoreductase

Note 1) Xanthine dehydrogenase (XDH) is one form (the dehydrogenase form) of xanthine oxidoreductase (XOR), which exists as XDH *in vivo*. Xanthine oxidase (XO) is also one form (the oxidase form) of XOR, which is converted from XDH under certain conditions. XOR inhibitors are described as "urate production inhibitors" in the Guideline for the Management of Hyperuricemia and Gout (2nd edition, in Japanese).

Note 2) In this Guideline, gene names are shown in italics, in accordance with the convention.

Introduction

Need for this Guideline and its Goals

Disease concept and research development on renal hypouricemia

Renal hypouricemia is a form of hypouricemia caused by a dysfunction of renal urate reabsorption. It does not include congenital purine metabolism abnormalities, including xanthinuria and purine nucleoside phosphorylase (PNP) deficiency, or secondary hypouricemia such as types caused by malignant tumors.

One of the characteristics of this disease is a low serum uric acid level. A case study of one patient showing hypouricemia was first reported by Praetorius *et al.*[1] in 1950. Greene *et al.*[2] reported a case study in 1972 on the characteristics of "hereditary disorder of urate reabsorption at the renal tubules," and Akaoka *et al.*[3] first reported it in Japan in 1975. It was known to be common in Japanese and Jewish populations from the beginning[4], and studies in Japan have contributed much to the understanding of this disease. For example, Ishikawa *et al.*[5, 6] named the concomitant kidney injury seen with renal hypouricemia (exercise-induced acute kidney injury) "ALPE" (Acute renal failure with severe Loin pain and Patchy renal ischemia after anaerobic Exercise) from its symptoms, and have researched it in detail. Moreover, causative genes (described below)[7,8] were identified in Japanese patients by Japanese researchers. Japanese researchers have made major contributions to studies on the development on this disease.

Classification of renal hypouricemia

Serum urate is excreted from the renal glomeruli to the tubules as primary urine, which is then reabsorbed via urate transporters, including URAT1/SLC22A12 and GLUT9/SLC2A9, which are expressed on the proximal tubular epithelium. Dysfunctions of these transporters cause renal hypouricemia. One nonfunctional variant, W258X, of *URAT1/SLC22A12* gene and two nonfunctional variants, R198C and R380W of the *GLUT9/SLC2A9* gene, have been identified as causes of renal hypouricemia. Renal hypouricemia caused by variants of the *URAT1/SLC22A12* gene and *GLUT9/SLC2A9* gene is respectively termed renal hypouricemia type 1 (MIM: 220150) and renal hypouricemia type 2 (MIM: 612076).

Most cases of renal hypouricemia have been discovered by chance in health examinations that detect low serum uric acid level. Clinically, renal hypouricemia shows a relationship with a high risk of urolithiasis and exercise-induced acute kidney injury.

The need to develop a guideline

Previous research suggests that renal hypouricemia is found in about 0.2% - 0.5% of the general Japanese population. The disease concept of renal hypouricemia is, however, not fully established, even in general clinical practice and health examination settings. It is rare for renal hypouricemia to be treated before any symptoms appear, and its medical treatment depends on individual healthcare providers' decisions. Well-defined standards and concrete guidance are therefore needed before appropriate medical practice, including diagnosis and treatment, can be provided. This state of affairs prompted us to develop this clinical practice Guideline for renal hypouricemia, to provide clear diagnostic guidance, to improve recognition of the disease, and to assist with making clinical decisions. We hope that our efforts will assist clinicians to gain a correct picture of the nationwide prevalence of renal hypouricemia and to promote a more accurate understanding of it, such as by researching new variants other than type 1 and type 2. These points are the fundamental reasons behind the need to develop this Guideline.

Goals

The first goal is to clarify the criteria for diagnosing renal hypouricemia, a prime requirement for trouble-free diagnosis of renal hypouricemia; and to enable appropriate decision-making, based on the results of blood examinations, as well as the selection of additional tests that will lead to a rapid and definitive diagnosis. Another goal is to work towards a consensus on clinical decision-making: the effects of drugs, including xanthine oxidoreductase inhibitors, and their application to the treatment of renal hypouricemia have up to now been controversial.

The use of a clinical practice Guideline should enable healthcare providers to remain informed and to gain a fuller picture of the status of renal hypouricemia. Various analyses and feedback to clinical settings based on this Guideline will also contribute to a more accurate understanding of the disease.

Why does the pathophysiology in peripheral blood and tubules of renal hypouricemia result in clinical symptoms such as exercise-induced acute kidney injury and urolithiasis? How is a sudden burden placed on the kidneys during strenuous exercise, and how can we counteract it? Research in these areas must be performed to acquire sufficient evidence. Trials must also be conducted to elucidate the mechanism of action of drugs, the benefits obtained by administering them, and whether or not adverse effects occur. Appropriate prevention of concomitant diseases is dependent on first clarifying their frequencies, risk factors, and conditions. Such prevention is one of the major goals of this guideline.

Hopes for the future

As described above, we hope that making this Guideline available to healthcare providers and individuals involved, including patients, will help to reveal the actual prevalence of renal hypouricemia, and lead to the discovery of novel genetic factors other than type 1 and type 2, especially in Japan, where the frequency of this disease appears to be relatively high. We also hope that this Guideline will encourage the development of novel therapies and preventive methods, not only for patient groups whose diagnosis and treatment are relatively well known, but also for those for whom truly effective treatments remain to be discovered.

We have developed this Guideline based on the "MINDS Manual for Guideline Development" to secure the largest possible number of scientific bases. I am grateful to all the Guideline Development Committee Members, who I hope will accept our heartfelt thanks again for all their help, and to those at the MINDS Guideline Center from whose advice we benefited.

References

1. Praetorius, E. & Kirk, J. E. Hypouricemia: with evidence for tubular elimination of uric acid. *J. Lab. Clin. Med.* **35**, 865-868 (1950).
2. Greene, M. L. *et al.* Hypouricemia due to isolated renal tubular defect. Dalmatian dog mutation in man. *Am. J. Med.* **53**, 361-367 (1972).
3. Akaoka, I. *et al.* Familial hypouricaemia due to renal tubular defect of urate transport. *Ann. Clin. Res.* **7**, 318-324 (1975).
4. Suzuki, T. *et al.* Genetic heterogeneity of familial hypouricemia due to isolated renal tubular defect. *Jinrui Idengaku Zasshi* **26**, 243-248 (1981).
5. Ishikawa, I. Acute renal failure with severe loin pain and patchy renal ischemia after anaerobic exercise in patients with or without renal hypouricemia. *Nephron* **91**, 559-570 (2002).
6. Ishikawa, I. ALPE. *Gout Nucleic Acid Metab.* **34**, 145-157 (2010) (in Japanese).
7. Enomoto, A. *et al.* Molecular identification of a renal urate anion exchanger that regulates blood urate levels. *Nature* **417**, 447-452 (2002).
8. Matsuo, H. *et al.* Mutations in glucose transporter 9 gene SLC2A9 cause renal hypouricemia. *Am. J. Hum. Genet.* **83**, 744-751 (2008).

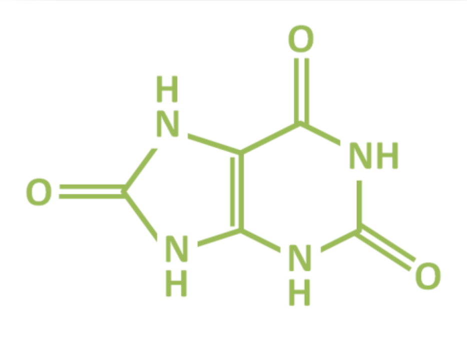

Methods Used to Develop this Guideline

1. Scope, population (patients and facilities) to whom the Guideline is meant to apply, and caveats

The scope of the Clinical Practice Guideline for Renal Hypouricemia (Guideline) covers two areas: (1) supporting the making of clinical decisions on the diagnosis of renal hypouricemia (RHUC), and on treatment plans for RHUC as well as methods of preventing its concomitant diseases; and (2) to enlighten healthcare personnel about RHUC.

The populations to whom the Guideline is intended to apply are Japanese (excluding infants) suspected of having RHUC due to low serum uric acid (S_{UA}), and RHUC patients. Secondly, and no less important, the intended users of the Guideline are healthcare personnel, especially those at first or second medical administration facilities (i.e., small-scale or mid-scale medical facilities) in Japan. The Guideline Development Committee (hereinafter "Committee") carefully summarizes the clinical information and points of note concerning RHUC, but the Committee does *not* intend that all users should apply every portrayal and recommendation in the Guideline uniformly to all patients, since clinical decision-making is fundamentally conducted under agreements between healthcare personnel and patients.

The Guideline is honored to include the comments of one representative RHUC patient. The Committee is planning to add the latest evidence on RHUC as well as to feed through patients' wishes to the Guideline in future editions.

2. The Committee and Conflicts of Interest (COIs)

All the Committee members are listed here. They are all members of Japanese Society of Gout and Nucleic Acid Metabolism (JSGNAM).

The Committee recorded all members' COIs from Oct. 1st, 2012 to Feb. 17th, 2017 (two years before developing the Guideline and up to its completion), if associated with organizations related in any way with the content of Guideline, under the criteria shown below.

(1) In an executive position or employed in an advisory capacity (more than one million yen/year; the Committee member or any of their family members)

(2) Owning stock (more than one million yen/year; the Committee member or any of their family members)

(3) Paid patent royalties (more than one million yen/year; the Committee member or any of their family members)

(4) Paid lecture fees (more than one million yen/year; the Committee member)

(5) Paid manuscript fees (more than one million yen/year; the Committee member)

(6) Receiving research funds (including fiduciary or collaborative research funds) (more than one million yen/year; the Committee member)

(7) In possession of scholarships or incentive donations (more than one million yen/year; the Committee member)

(8) Having an endowed chair (full-time position or not)

(9) Other items of note (more than one million yen/year; the Committee member or any of their family members).

For the disclosure of COIs, a name list is shown of organizations for which each Committee member has self-reported under the above criteria. The details of individual reports are not disclosed.

The list below addresses the COIs of any of the Committee members.

(1) None

(2) None

(3) None

(4) Teijin Pharma Ltd, Sanwa Kagaku Kenkyusho Co., Ltd. Fujiyakuhin Co., Ltd.

(5) None

(6) Teijin Pharma ltd, Sanwa Kagaku Kenkyusho Co., Ltd. Fujiyakuhin Co., Ltd.

(7) Teijin Pharma ltd, Sanwa Kagaku Kenkyusho Co., Ltd., Fujiyakuhin Co., Ltd, Torii Pharmaceutical Co., Ltd.

(8) None

(9) None

For the reported COIs shown above, the Committee has devised the following measures.

- A member with a COI cannot be a leader of the Committee.
- The total number of members with a COI cannot exceed two thirds of all members.
- Members with a COI may not take charge of systematic reviews of items associated with the interest(s) in question.
- The Delphi Method is adopted for decision-making on recommendations to block any influence of the interests of certain Committee members.

Furthermore, the Committee considers that the leader of the Committee should clarify his COIs, because he supervises and overviews the development of the Guideline. In this case, the Committee member must clearly declare that he has no COIs of the types described above in (1) to (9).

Based on the COI information above, the Committee decided to share out the members' roles as described below. Team leaders are underlined.

- Guideline Supervising Committee

  <u>Shinomiya</u>, Hisatome, Ichida

- Guideline Developing Group (GDG)

  <u>Ichida</u>, Matsuo, Hakoda, Yamaguchi, Nakayama

- Systematic Review Team (SRT)

  <u>Shinomiya</u>, Hosoyamada, Ogino, Hamada, Ohtahara

- Guideline Secretariat

  <u>Matsuo</u>, Nakayama

3. Funding

The development of the Guideline was exclusively funded by a proportion of a grant provided for "Research to Develop Diagnostic Criteria/clinical Practice Guidelines on Rare/intractable Diseases of the Kidney and Urinary Systems (Lead researcher: Kazumoto Iijima)," as one theme of "Research on Measures

for Intractable Diseases” funded by a Health and Labour Sciences Research Grant provided by the Ministry of Health, Labour and Welfare of Japan (fiscal year: 2014 - 2016).

4. Methods of developing the Guideline

1. MINDS Manual for Guideline Development (Yamaguchi, N. *et al.*, Ver. 1.0 (2014) or Yamaguchi, N. *et al.*, Ver. 1.1 (2014) or Kojimahara, N. *et al.*, Ver. 2.0 (2016), Tokyo, Japan Council for Quality Health Care) was referred to for developing the Guideline.

2. The RHUC research team invited JSGNAM to co-edit the Guideline in July 2014. This was granted approval by JSGNAM’s Executive Board in September 2016. The Committee was then organized jointly. All the Committee members attended the training workshops organized by the Japan Council for Quality Health Care, or viewed its video lessons.

3. Communications were chiefly conducted by e-mail. Regular conferences, shown below, were also held to maintain close coordination and to discuss important matters. First conference: Keio Plaza Hotel, Tokyo, on

Table 1 Settings for Clinical Question 1

<table>
<tr><th colspan="4">Key Clinical Issue</th></tr>
<tr><td colspan="4">Key Clinical Issue 1: Diagnosis of renal hypouricemia (RHUC)
Hypouricemia is a clinical finding often found by chance during health examinations. One pathognomonic finding of RHUC is a low serum uric acid (S_{UA}) level, although the threshold for suspecting RHUC has not been determined: so far, for example, the definition of hypouricemia is $S_{UA} \leq 2$ mg/dl in some studies and $S_{UA} \leq 3$ mg/dl in others. A clear cutoff value for diagnosing RHUC, one cause of hypouricemia, is therefore necessary.</td></tr>
<tr><th colspan="4">Components of CQ</th></tr>
<tr><th colspan="4">P (Patients, Problem, Population)</th></tr>
<tr><td>Sex</td><td colspan="3">Not specified</td></tr>
<tr><td>Age</td><td colspan="3">Excluding infants</td></tr>
<tr><td>Problem</td><td colspan="3">Hypouricemia (before definite diagnosis of RHUC)</td></tr>
<tr><td>Physiographic factor</td><td colspan="3">Not specified</td></tr>
<tr><td>Others</td><td colspan="3">Centrally focused on Japanese individuals</td></tr>
<tr><th colspan="4">List of I (Interventions) or C (Comparisons, Controls, Comparators)</th></tr>
<tr><td colspan="4">➢ To consider the differential diagnosis of hypouricemia when his/her S_{UA} is ≤ 2 mg/dl.
➢ To consider the differential diagnosis of hypouricemia when his/her S_{UA} is ≤ 3 mg/dl.
➢ To consider the differential diagnosis of hypouricemia when his/her S_{UA} is ≤ 4 mg/dl.</td></tr>
<tr><th colspan="4">List of O (Outcomes)</th></tr>
<tr><th></th><th>Outcome</th><th>Benefit or Harm</th><th>Magnitude (points)</th></tr>
<tr><td>O1</td><td>To obtain valuable findings to suspect RHUC</td><td>Benefit</td><td>8</td></tr>
<tr><th colspan="4">Developed CQ</th></tr>
<tr><td colspan="4">CQ1: Should individuals with a serum uric acid level of ≤ 2.0 mg/dl be considered for differential diagnosis of hypouricemia?</td></tr>
</table>

on February 19th, 2015.

Second conference: Senri Life Science Center, Osaka, on February 19th, 2016.

Third conference: Keio Plaza Hotel, Tokyo, on February 17th, 2017.

4. GDG members drew up the Scope, Key Clinical Issues, and Clinical Questions (CQ). These were confirmed and approved at the first conference (**Table 1**, **Table 2**: The "outcome" in these Tables describes only the finally-adopted ones for CQs. The "magnitude" was evaluated on a scale of 1 to 10 points).

5. SRT conducted a literature search using the following methods.

- Objectives: For individual research papers, the Medline/PubMed, Igaku Chuo Zasshi (the Japan Medical Abstracts Society) databases were searched. For systematic reviews or meta-analysis papers, Medline/PubMed, The Cochrane Library (including The Cochrane Database of Systematic Reviews (CDSR)), UpToDate, and The Database of Abstracts of Reviews of Effects (DARE) were investigated. Only papers in English or Japanese were searched.
- Time period: From 1966 to December 31st, 2014.

Table 2 **Settings for Clinical Question 2**

Key Clinical Issue			
**Key Clinical Issue 2: Prevention plan for the concomitant diseases of renal hypouricemia (RHUC)**<br>RHUC patients often suffer from recurrent concomitant diseases, such as exercise-induced acute kidney injury (EIAKI) and urolithiasis. To prevent these concomitant diseases, patients are usually recommended to drink a lot (especially before exercise), to avoid intense exercise, and/or to limit exercise when taking non-steroid anti-inflammatory drugs. Moreover, in addition to using urinary alkalinizers to prevent urolithiasis, administration of xanthine oxidoreductase (XOR) inhibiters before exercise is also assumed to prevent EIAKI. The clinical effectiveness of XOR inhibitors, however, remains unclear and needs to be investigated.			
**Components of CQ**			
**P (Patients, Problem, Population)**			
**Sex**	Not specified		
**Age**	Excluding infants		
**Problem**	RHUC		
**Physiographic factor**	Not specified		
**Others**	Centrally focused on Japanese individuals		
**List of I (Interventions) or C (Comparisons, Controls, Comparators)**			
➢ XOR inhibitors (Allopurinol, Febuxostat, and Topiroxostat)<br>➢ Other drugs<br>➢ No administration			
**List of O (Outcomes)**			
	**Outcome**	**Benefit or Harm**	**Magnitude (points)**
**O1**	Development/recurrence of EIAKI	Harm	10
**O2**	Adverse events caused by XOR inhibitors	Harm	7
**Developed CQ**			
**CQ2: Should XOR inhibitors be administered to prevent EIAKI in patients with RHUC?**			

- Types of evidence: Systematic review papers were given priority followed by meta-analysis papers and individual research papers. Investigated in the category of individual research papers were randomized controlled studies, non-randomized controlled studies, and observational studies. If the search results turned up no papers with a high enough evidence level, case reports were also investigated.
- Policy on literature searching: the PICO format was used for literature searching (**Table 1**, **Table 2**: The "outcome" in these Tables describes only finally-adopted ones for CQs). Both "P" and "I" are set for searching, and "C" is also used when necessary. "O" is not set. Two independent SRT members in charge of each CQ conducted literature searching and screening. Literature that was not searched systematically could be included in the screening results if the member in charge considered them appropriate. The searched literature was subsequently screened by reading its titles and abstracts to narrow them down to meet each CQ (1st screening). All literature identified by two SRT members was collated by another member, and each member repeated screening again by reading all the manuscripts (2nd screening). The results were collated again and adopted as the results of the systematic review for each CQ.
- Other databases: To search for established guidelines for RHUC, the National Guideline Clearinghouse (NGC), NICE Evidence Search, International Guideline Library, and Minds Guideline Center databases were investigated on June 1st, 2015. However, no relevant material was found. Furthermore, to search clinical studies on RHUC, the Grey Literature Report, OpenGrey, ClinicalTrials.gov, Virtual Health Library, World Health Organization (WHO), UMIN Clinical Trials Registry, and National Institute of Public Health Clinical Trial Search databases were investigated on June 1st, 2015. No relevant material was found.

6. The results of the literature search are as below.

545 papers were found, of which 455 were adopted after screening for CQ1, and 43 of 54 papers passed CQ2. All had a low evidence level, comprising case reports, cross-sectional studies, or experts' opinions. SRT members made lists of PICO, etc., from this literature, and a systematic review report was compiled based on the list.

7. GDG conclusively decided on recommendations and their strength at the Second Conference, based on the submitted systematic review report by SRT. The Delphi method had been selected as the consensus building system for recommendations and their strength, although it was not used due to pre-existing unanimous approval. The draft version of the Guideline was developed

based on this approval.

8. Because one of the aims of the Guideline is to enlighten healthcare personnel on RHUC, textbook descriptions were also added by all Committee members in addition to CQ. All these description were mutually peer-reviewed.

There is no research on RHUC with sufficiently good evidence that is appropriate for inclusion in either systematic reviews or clinical practice guidelines. However, it is also true that there is no sufficient foundation on which to prepare high-quality intervention studies on RHUC, taking into account the fact that RHUC itself is not a common disease and that the pathophysiology of RHUC has yet to be elucidated. Therefore, excluding all research reports on RHUC without exception due to low evidence levels would send the wrong message to clinical practitioners, and would also deviate from the aims of the Guideline. The Committee then decided to add “Consensus levels” as expert opinions on each statement in the textbook description. Consensus levels were defined according to the median values of all Committee members’ evaluations, on a scale of 1 - 5 (1 = “disagree,” 3 = “neutral,” and 5 = “agree”).

9. The results of external reviews are described below.

Prior to publication of the Guideline, the Committee commissioned an evaluation of the Guideline draft by an external reviewer (not a member of JSGNAM) using the AGREE II method (AGREE Next Steps Consortium 2009: http://www.agreetrust.org/) with the support of The Japanese Society for Pediatric Nephrology. The COIs were sourced for the external reviewer subject to the same standard as for Committee members. The external reviewer declared no COIs. A summary of the comments and correspondences is as follows.

a. To show the populations to whom the Guideline is meant to apply.

• We showed the intended population.

b. It was not possible to secure a total lack of bias on development Guidelines, since all the Guideline Development Committee Members are JSGNAM members.

• We established and described the task of cooperating with related academic societies for future dissemination and revision of the Guideline.

c. To clarify the facilitators and barriers to the Guideline’s application.

• We described them.

d. To provide tools on how the recommendations can be put into practice.

• We provided “A Brief Summary of this Guideline” as an abstract.

Public comments were also solicited via the GUIDE system provided by MINDS (http://minds.jcqhc.or.jp/guide/pages/GuideTopHome.aspx) for the month from Nov. 8th to Dec. 8th 2016. We found no comments requesting revisions or modifications.

In the light of these comments (or lack of them), the finalized Guideline was approved and completed at the Third Conference.

The Guideline draft was also read through and evaluated by an RHUC patient, and her comments were added to the Guideline as “Notes from an Athlete.”

10. We considered the facilitators and barriers to the Guideline’s application to be as follows.

The first of the two recommendations in the Guideline states, “We strongly recommend that individuals who have an S_{UA} level of ≤ 2.0 mg/dl (120 μmol/l) be considered for differential diagnosis of hypouricemia.” A factor that eases the application of this recommendation is that a distinct threshold is provided for S_{UA} itself, which is normally measured during common medical examinations. On the other hand, a barrier factor is that the young population has fewer opportunities to be examined for their S_{UA} level, resulting in a lower likelihood of possible RHUC being detected. To overcome this problem, it is possible to recommend that their S_{UA} level be evaluated during regular school medical examinations, alongside tests for anemia. But this would be still premature, since the precise facts about this disease, such as its rate of prevalence, are currently not known.

In the second recommendation, we state that we cannot definitively state that XOR inhibitors should be administered to prevent EIAKI in RHUC patients. However, it is possible that XOR inhibitors might prevent the onset or relapses of EIAKI. Administration of XOR inhibitors should therefore be decided in the light of its potential benefits and harms, especially for athletes and high-risk patients with a past history of EIAKI attacks. The upside to this recommendation is that it elucidates the fact that both benefits and harms exist, whereas the barrier factor is that this recommendation does not clearly recommend the direction of the clinical decision. To enable a clear recommendation is a future research task: we assume that evidence on this question will be compiled.

11. Efforts after publication are as follows.

After publication, the Committee conducts public relations activities via JSGNAM meetings and the internet to promote adoption of the Guideline in daily clinical practice. The Committee plans to revise the Guideline three to five years after initial publication, and also to continue collecting evidence through regular literature searches, and to obtain novel findings on RHUC by conducting research meetings to discuss the next revision. By means of these programs, the Committee will investigate the need for revisions to the Guideline as well as start recruitment of new Committee members to effect revisions. We also set it as a task

to cooperate with academic societies with medical care and research programs, for future dissemination and revision of the Guideline.

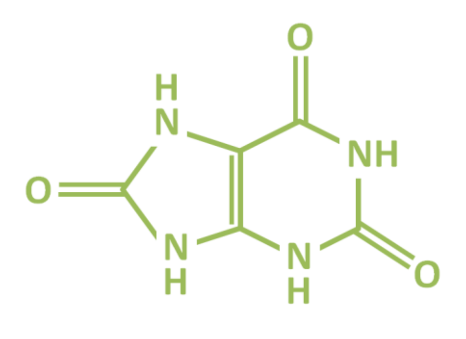

1 Clinical Algorithm for Renal Hypouricemia

Clinical Algorithm for Renal Hypouricemia (RHUC)

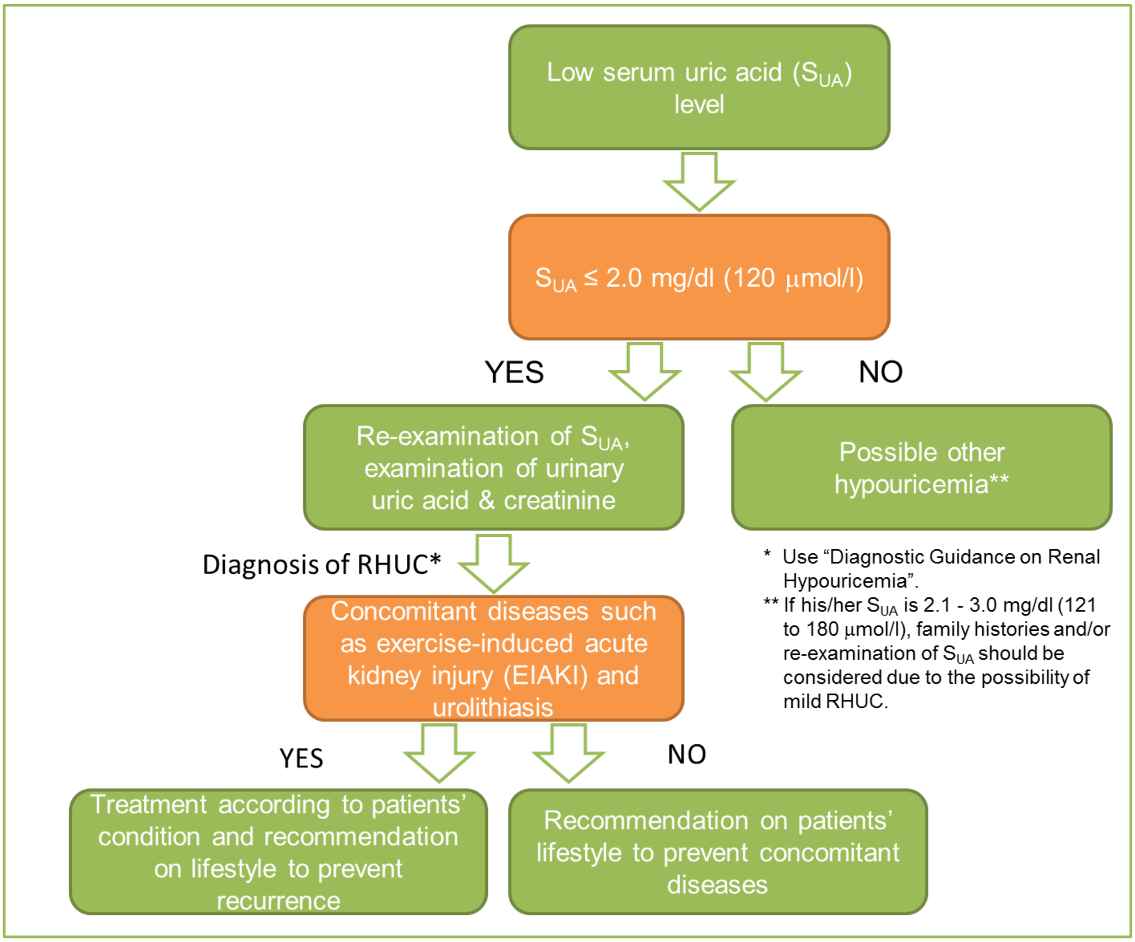

2

The Epidemiology of Renal Hypouricemia

Statement

1.	The prevalence of renal hypouricemia is estimated to be approximately 0.2% in males and 0.4% in females in Japan. Consensus 4 ★★★★☆
2.	Concomitant diseases of renal hypouricemia include urolithiasis and exercise-induced acute renal kidney injury, but their frequencies need to be accurately elucidated. Consensus 4 ★★★★☆

Summary

If hypouricemia is defined as a serum uric acid (S_{UA}) level of ≤ 2.0 mg/dl (120 µmol/l), the prevalence of renal hypouricemia (RHUC) can be estimated to be approximately 0.2% in males and 0.4% in females. In females, the prevalence might be higher for individuals before menopause. Urolithiasis and exercise-induced acute renal kidney injury (EIAKI) are known to be concomitant diseases of RHUC, but their frequencies are not known. Recent studies have reported that vascular endothelial dysfunction and lowered glomerular filtration are also concomitant with RHUC.

1. Prevalence of RHUC

RHUC is defined as hypouricemia caused by increased renal urate excretion. In contrast to the definition of hyperuricemia, which is based on the serum solubility of sodium urate, no distinct standard for hypouricemia has yet been established. An S_{UA} of ≤ 2.0 mg/dl has up to now been commonly used as the definition of hypouricemia[1,2]. With this definition, the study on the prevalence of hypouricemia by Wakasugi *et al.*[3] is the largest and most representative of the general Japanese population: the number of subjects analyzed was 90,170 males and 136,935 females, using S_{UA} data from participants undergoing governmental Specific Health Checkups in 24 prefectures. According to their report, 193 males (0.21%) and 540 females (0.39%) had hypouricemia. Tabe[4] investigated the results for individuals who had undergone comprehensive medical examinations (MEs) and in-company health checkups (17,603 males and 3,544 females in total), and reported that the frequency of those with an S_{UA} of ≤ 2.0 mg/dl was 0.14% for males and 0.40% for females. Kaneko *et al.*[5] also examined around 10,000 comprehensive ME participants every year for four years, and discovered that 0.09 - 0.12% of males and 0.36 - 0.51% of females had hypouricemia. Matsuo *et al.*[6] studied over 21,260

Japanese military (Self-Defense Forces) personnel and reported that 0.18% showed an S_{UA} of ≤ 2.0 mg/dl. Although the exact percentage was not known, male participants were in the majority in that study since the subjects were all military personnel. As described above, data from large-scale studies indicate that, in Japan, the frequency of hypouricemia is approximately 0.2% in males and 0.4% in females.

Almost all cases of hypouricemia are thought to have originated from RHUC.[2,4] However, Hisatome *et al.*[7] reported that while 13 out of 3,258 outpatients showed hypouricemia, eight showed transient hypouricemia caused by other diseases (diabetes mellitus, liver cirrhosis, and hepatic carcinoma) or drugs (allopurinol and benzbromarone). Since the subjects of the study by Wakasugi *et al*.[3] are population-based residents aged 40 - 74 years, the frequency of those with concomitant diseases should be lower than that in the study by Hisatome *et al*.[7] In fact, the frequency of patients of diabetes mellitus was not higher in those with hypouricemia than in those without[3]. Although there was no information provided on liver disease, the averages of AST, ALT, and γ-GTP in hypouricemic subjects did not differ from those in the SUA > 2.0 mg/dl groups[3]. There was also no information on the use of urate-lowering agents, but there were no differences in the frequency of hypouricemia among different age groups in males[3], whereas increasing numbers of males receive frequent urate-lowering therapy as they age[8]. As described above, the prevalence of hypouricemia reported by Wakasugi *et al.*[3] is thought to reflect the prevalence of RHUC.

2. Sexual differences in the prevalence of RHUC

As described above, the prevalence of hypouricemia in females is about double that seen in males. Wakasugi *et al.*[3] first investigated the prevalence of hypouricemia with age, and revealed that its prevalence decreased with rising age in females (**Fig. 1**). The age-dependent decrease in the prevalence of hypouricemia in females seems to have been due to the menopause, which causes a loss in the urate-lowering effect of female sex hormones. The rate of prevalence of hypouricemia in females in their 40s was 0.6% (**Fig. 1**). Although menopausal status was not investigated in the study by Wakasugi *et al.*,[3] the female population in their 40s is likely to have experienced menopause, because the onset of menopause, in 80% of Japanese females, is from age 45 - 54. Therefore, its prevalence is likely to show a higher result if only the female population before menopause is investigated.

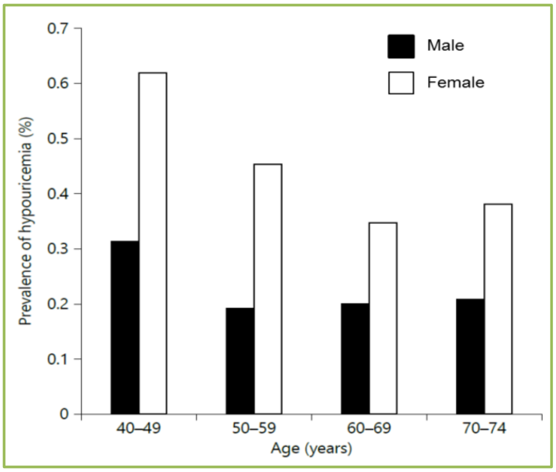

Fig. 1 The prevalence of hypouricemia
(Drawn from Reference 3)

It seems unlikely that the *URAT1/SLC22A12* gene variants, which are the cause of most cases of RHUC,[9–11] are distributed differently among males and females, since the gene is located on an autosome. Therefore, the reason for the higher frequency of hypouricemia seen in females, especially in pre-menopausal individuals, would be because the effects of *URAT1/SLC22A12* gene variants on the S_{UA} levels are more likely to be enhanced through the urate-lowering effects of female sex hormones. In fact, Ichida *et al.*[11] reported that seven of eleven hypouricemia cases with heterozygous variants were females (S_{UA} levels of heterozygotes in *URAT1/SLC22A12* gene variants are higher than those in homozygous cases). Sugihara *et al.*[12] found that all of five heterozygous hypouricemic cases were females.

3. Frequency of concomitant diseases of RHUC

Urolithiasis is one of the concomitant diseases of RHUC, but its frequency has not been investigated in large-scale studies. Ichida *et al.*[11] investigated 71 RHUC cases and found that six (8.5%) had experienced urolithiasis: four (9.3%) of 43 males and two (7.1%) of 28 females. A research group identified 179 RHUC cases in a nationwide questionnaire study and found that 11 (6.1%) had had urolithiasis[13]. A nationwide survey of urolithiasis conducted by The Japanese Urological Association in 2005[14] revealed that its yearly incidence was 0.19% in males and 0.08% in females, with lifetime incidence being 15.1% in males and 6.8% in females. There is no data on the yearly incidence or lifetime incidence of urolithiasis in patients with RHUC.

EIAKI is also one of the pathognomonic concomitant diseases of RHUC, but its precise frequency remains to be revealed. Ichida *et al.*[11] reported that 15 (21.1%) of 71 RHUC cases had experienced EIAKI, with all of them being male, indicating its prevalence to be 34.9% in 43 male RHUC cases. This frequency seems to be fairly high, but it may be partly because the study population consisted of patients at a university hospital who already tended to have a complex pathogenic status. Another study by Ichida *et al.*[10] revealed that the frequency of EIAKI was 6.5% (two of 31) in RHUC patients if those diagnosed with RHUC were excluded based on the onset of EIAKI. Ishikawa[15] examined EIAKI cases with normal or slightly elevated serum levels of creatine kinase and myoglobin, and showed that of 96 cases for whom S_{UA} data were available, about half (49 cases: 51%) had been diagnosed with RHUC. A nationwide questionnaire[13] showed that 11 (6.1%) of 179 RHUC cases experienced EIAKI.

Sugihara *et al.*[12] reported vascular endothelial dysfunction in RHUC patients. This is a vascular-endothelial-dependent vasodilation response. Its dysfunction increases the risk of atherosclerosis. They showed that vascular endothelial dysfunction was frequently observed in individuals with $S_{UA} \leq 0.8$ mg/dl, and that all of such cases were homozygous for *URAT1/SLC22A12* variants. Wakasugi *et al.*[3] reported the association between hypouricemia and decreased glomerular filtration rates in males but not in females. Tabara *et al.*[16] also reported that a nonsense mutation of the *URAT1/SLC22A12* gene which causes hypouricemia was associated with decreased glomerular filtration rates. Therefore, although

RHUC might be implicated as a cause of renal dysfunction, further accumulation of epidemiological data and investigations of the mechanism for this association are needed.

References

1. Ohta, T. *et al.* Exercise-induced acute renal failure associated with renal hypouricaemia: results of a questionnaire-based survey in Japan. *Nephrol. Dial. Transplant.* **19**, 1447- 1453 (2004).
2. Ichida, K. Renal hypouricemia. *Hyperuricemia Gout* **17**, 28- 32 (2009) (in Japanese).
3. Wakasugi, M. *et al.* Association between hypouricemia and reduced kidney function: a cross-sectional population-based study in Japan. *Am. J. Nephrol.* **41**, 138-146 (2015).
4. Tabe, A. Research on the pathophysiology of hypouricemia. *Tokyo Jikei-kai Ika Daigaku Zasshi* **111**, 821- 839 (1996) (in Japanese).
5. Kaneko, K. & Fujimori, S. Hypouricemia: frequency and clinical significance of low uric acid level. *Medical Practice* **12**, 659-662 (1995) (in Japanese).
6. Matsuo, H. *et al.* Mutations in glucose transporter 9 gene SLC2A9 cause renal hypouricemia. *Am. J. Hum. Genet.* **83**, 744- 751 (2008).
7. Hisatome, I. *et al.* Cause of persistent hypouricemia in outpatients. *Nephron* **51**, 13-16 (1989).
8. Hakoda, M. & Tomita, M. Frequency differences in age in hyperuriceia—analyses using a medical fee receipt database. *Gout Gout Nucleic Acid Metab*. **37**, 111-116 (2013) (in Japanese).
9. Enomoto, A. *et al.* Molecular identification of a renal urate anion exchanger that regulates blood urate levels. *Nature* **417**, 447-452 (2002).
10. Ichida, K. *et al.* Clinical and molecular analysis of patients with renal hypouricemia in Japan-influence of URAT1 gene on urinary urate excretion. *J. Am. Soc. Nephrol.* **15**, 164-173 (2004).
11. Ichida, K. *et al.* Age and origin of the G774A mutation in SLC22A12 causing renal hypouricemia in Japanese. *Clin. Genet.* **74**, 243-251 (2008).
12. Sugihara, S. *et al.* Depletion of uric acid due to SLC22A12 (URAT1) loss-of-function mutation causes endothelial dysfunction in hypouricemia. *Circ. J.* **79**, 1125-1132 (2015).
13. Shinomiya, N. Studies to Reveal the Incidence of Renal Hypouricemia Nationwide. *Final report for a Health and Labour Sciences Research Grant* (2014) (in Japanese).
14. Yasui, T. *et al.* Prevalence and epidemiological characteristics of urolithiasis in Japan: national trends between 1965 and 2005. *Urology* **71**, 209-213 (2008).
15. Ishikawa, I. Acute renal failure with severe loin pain and patchy renal ischemia after anaerobic exercise in patients with or without renal hypouricemia. *Nephron* **91**, 559-570 (2002).
16. Tabara, Y. *et al.* Association of four genetic loci with uric acid levels and reduced renal function: the J-SHIPP Suita study. *Am. J. Nephrol.* **32**, 279-286 (2010).

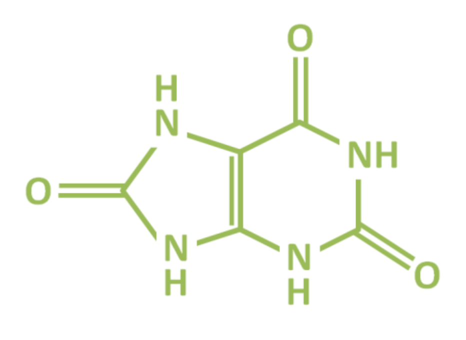

3 The Pathophysiology of Renal Hypouricemia

Statement

1.	Renal hypouricemia has the characteristics of low serum uric acid level, and is caused by increased renal urate excretion.	Consensus 5 ★★★★★
2.	Renal hypouricemia caused by variants of urate reabsorption transporter genes *URAT1/SLC22A12* and *GLUT9/SLC2A9* is termed "renal hypouricemia type 1" and "type 2," respectively.	Consensus 5 ★★★★★
3.	Renal hypouricemia types 1 and 2 themselves are usually asymptomatic, but exercise-induced acute renal kidney and urolithiasis are known as its concomitant diseases.	Consensus 5 ★★★★★

Summary

Renal hypouricemia (RHUC) has the characteristic of low serum uric acid (S_{UA}) level, and is caused by increased renal urate excretion due to decreased urate reabsorption at the renal proximal tubules. Urate is mainly reabsorbed at the renal proximal tubules via urate transporters encoded by the *URAT1/SLC22A12* gene and *GLUT9/SLC2A9* gene. Each nonfunctional variant causes renal hypouricemia type 1 (RHUC1) and type 2 (RHUC2). RHUC is notably observed in Japanese, with RHUC1 predominating. The W258X variant of *URAT1/SLC22A12* is commonest causative variant of RHUC1, followed by the R90H variant. Renal hypouricemia types 1 and 2 themselves are usually asymptomatic, but exercise-induced acute renal kidney and urolithiasis are known as complications. There are also cases of RHUC for which the causative genes are not known.

1. The metabolism and kinetics of urate

Purine bases such as adenine and guanine are components of nucleic acid and adenosine triphosphate (ATP). Although most mammals, including mice, are able to metabolize urate to allantoin via urate oxidase (uricase), hominoids, including humans and chimpanzees, cannot metabolize it due to a lack of uricase activity: the uricase gene has changed into a pseudo-gene during the evolutionary process.[1,2] Urate is therefore an end metabolite of purine bodies (**Fig. 1**). Humans normally produce about 700 mg/day of urate, mainly in the liver, two thirds of which is excreted from the kidney and the remaining one third from extra-renal pathways such as the intestine.

2. Physiological urate reabsorption and the pathophysiology of renal hypouricemia

All urate is filtered by the renal glomeruli, and

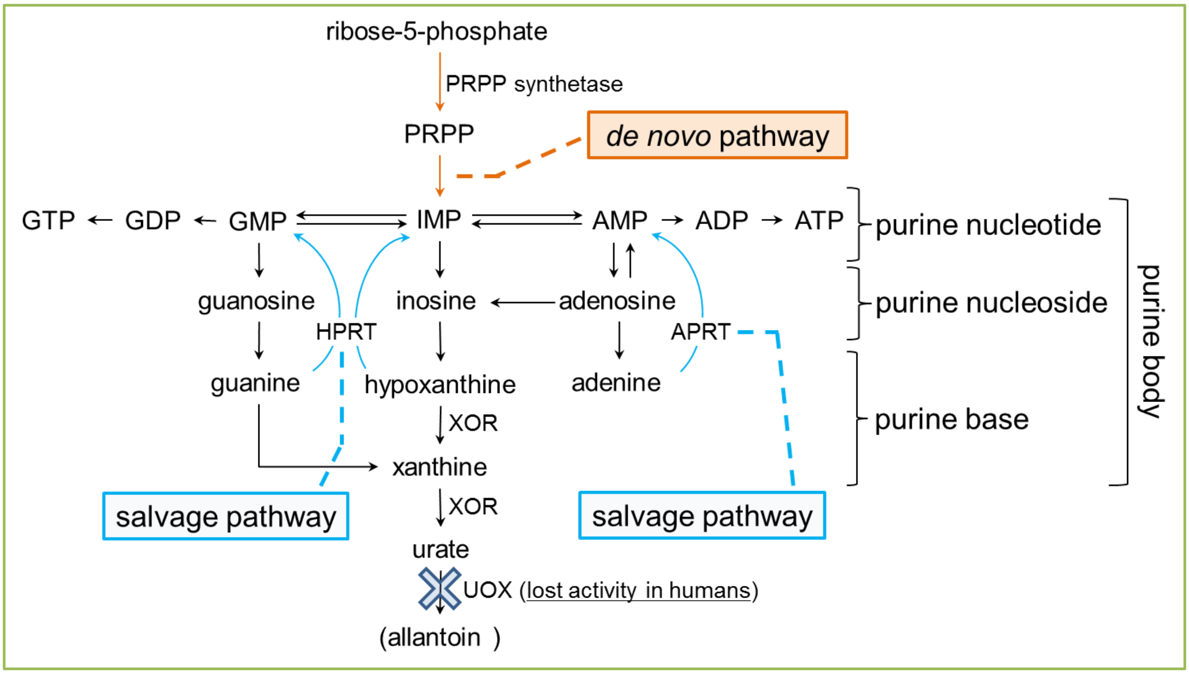

Fig. 1 Metabolism of urate

Urate is an end metabolite of purine bodies in humans. Purine bases are supplied from *de novo* pathways, which produce new purine bases, and are also supplied from salvage pathways that re-use purine bases. They are finally metabolized to urate by XOR, but humans cannot metabolize it due to a lack of uricase activity: the uricase gene has changed into a pseudo-gene during the evolutionary process. (See Abbreviations for details.)

almost all is reabsorbed at the proximal tubules. The finally excreted amount of urinary urate (approximately 6 - 10%) is chiefly dictated by the reabsorption efficiency of the proximal tubules. Urate transporters which reabsorb urate from the primary urine side to the blood vessel side through cell membranes are called urate reabsorption transporters (**Fig. 2A**). There is another urate transporter called the urate excretion transporter, which secretes urate from the blood vessel side to the primary urine side (tubular side). Dysfunctional variants of urate reabsorption transporter increase urate excretion (**Fig. 2B, C**), which results in lowered serum uric acid (S_{UA}) level: this is the basic pathophysiology of renal hypouricemia (RHUC).

The first report on this disease was published in 1950[3], and the first report from Japan was in 1975[4]. From the very beginning, this disease has been noted to be common in Japanese and Jews[5]. Subsequent studies identified the causative genes of RHUC[6,7], and its molecular pathophysiology is now almost fully known.

3. Causative genes of renal hypouricemia

In the human kidney, physiological urate reabsorption is mainly mediated via two urate reabsorption transporters. These are urate transporter 1 (URAT1/SLC22A12) and glucose transporter 9 (GLUT9/SLC2A9)[6–8] (**Fig. 2**). RHUC caused by genetic variants of *URAT1/SLC22A12* and *GLUT9/SLC2A9* is termed hypouricemia type 1 (RHUC1; MIM: 220150), and type 2 (RHUC2; MIM: 612076), respectively (**Table**)[9,10].

As shown in **Fig. 3**, both the causative variants of RHUC1 and RHUC2 are nonfunctional variants

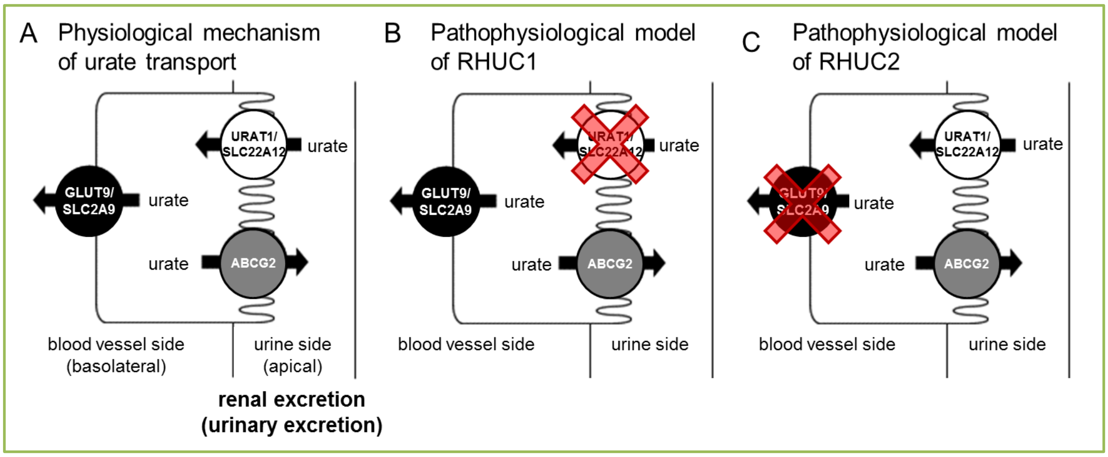

Fig. 2 Physiological mechanism of urate transport and pathophysiological model of renal hypouricemia at the proximal tubules

(A) Physiological mechanism of urate transport at human kidney. There are three representative transporters in human that regulate serum uric acid (S_{UA}) levels: two urate reabsorption transporters URAT1/SLC22A12 and GLUT9/SLC2A9, and one urate excretion transporter ABCG2.
(B) Pathophysiological model of renal hypouricemia type 1 (RHUC1). Decreased urate reabsorption function of URAT1/SLC22A12 causes increased urinary excretion as well as decreased S_{UA}.
(C) Pathophysiological model of renal hypouricemia type 2 (RHUC2). Decreased urate reabsorption function of GLUT9/SLC2A9 causes increased urinary excretion as well as decreased S_{UA}.

(Drawn from Reference 8)

for transportation of urate[6-8]. Exercise-induced acute renal kidney injury (EIAKI)[11,12] and urolithiasis[11] are known to be concomitant diseases of RHUC: they are observed in both RHUC1[13] and RHUC2[14] patients.

1) Renal hypouricemia type 1 (RHUC1)

URAT1/SLC22A12 is a gene that was first identified as a causative gene of RHUC in 2002.[6] The most frequent variant in Japanese RHUC patients is the loss-of-function W258X variant, in which the 258th amino acid, tryptophan (W) is mutated to a stop codon (X): rs121907892. The second most frequent variant is also a loss-of-function variant, R90H, in which the 90th amino acid, arginine, (R) is mutated to histidine (H): rs121907896. Having either W258X or R90H (heterozygotic), or both (homozygotic, or compound heterozygotic[Note)] has been reported to decrease mean SUA from 6.2 mg/dl to 4.0 mg/dl or 0.8 mg/dl in Japanese males, and from 4.5 mg/dl to 3.5 mg/dl or 0.6 mg/dl in Japanese females[15]. Therefore, there could be heterozygous cases of hypouricemia with an S_{UA} of 2.1 mg/dl to 3.0 mg/dl[7], that is, mild RHUC cases. There are also other variants, such as the E298D variant, in which the 298th amino acid, glutamine acid (E) is

Table 1 Human urate transporters and renal hypouricemia

Urate transporters	Locus	Physiological function (urate transport)	Urate-related disease caused by dysfunctional transporters
**URAT1/SLC22A12**	11q13	Urate reabsorption at renal proximal tubules	Renal hypouricemia type 1 (RHUC1)
**GLUT9/SLC2A9**	4p16-p15.3	Same as above	Renal hypouricemia type 2 (RHUC2)

(Drawn from Reference 10)

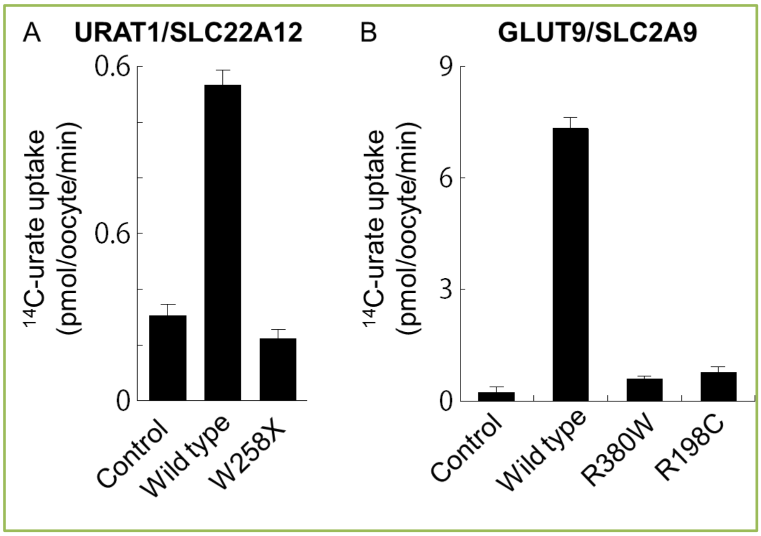

Fig. 3 Functional analysis of urate transport

The result of functional analysis of **(A)** URAT1/SLC22A12 and **(B)** GLUT9/SLC2A9. Each mutation causes a loss of function of urate transport. (Drawn from Reference 6 to 8)

mutated to asparagine (D): rs121907894[6]; and the T217M variant, in which the 217th amino acid threonine (T) is mutated to methionine (M): rs121907893)[6,13], although their frequency is low.

Note) A compound heterozygote means having two heterozygote causative variants (paternal- and maternal-derived variants) at two distinct loci on a certain gene.

2) Renal hypouricemia type 2 (RHUC2)

GLUT9/SLC2A9 is a urate reabsorption transporter gene that was the next to be identified as a causative gene of RHUC[7] through analyses of RHUC patients without variants of the *URAT1/SLC22A12* gene. While loss-of-function variants such as the R380W variant (the 380th amino acid arginine (R) is mutated to tryptophan (W): rs121908321) and the R198C variant (the 198th amino acid arginine (R) is mutated to cysteine (C): rs121908322), are reported[7], the total frequency of RHUC2, including by these variants, is much less common than that of RHUC1. The clinical data shows that while the fractional excretion of uric acid (FE_{UA}; see Chapter 4 for details) of RHUC1 cases are about 30 - 70%, those of RHUC2 are often over 100%, especially in cases with homozygous mutations[14].

Furthermore, there are still unidentified cases with causative genes other than RHUC1 and RHUC2.

References

1. Wu, X. W., Lee, C. C., Muzny, D. M. & Caskey, C. T. Urate oxidase: primary structure and evolutionary implications. *Proc. Natl. Acad. Sci. U. S. A.* **86**, 9412-9416 (1989).
2. Oda, M., Satta, Y., Takenaka, O. & Takahata, N. Loss of urate oxidase activity in hominoids and its evolutionary implications. *Mol. Biol. Evol.* **19**, 640-653 (2002).
3. Praetorius, E. & Kirk, J. E. Hypouricemia: with evidence for tubular elimination of uric acid. *J. Lab. Clin. Med.* **35**, 865-868 (1950).
4. Akaoka, I., Nishizawa, T., Yano, E., Takeuchi, A. & Nishida, Y. Familial hypouricaemia due to renal tubular defect of urate transport. *Ann. Clin. Res.* **7**, 318-324 (1975).
5. Suzuki, T., Kidoguchi, K. & Hayashi, A. Genetic heterogeneity of familial hypouricemia due to isolated renal tubular defect. *Jinrui Idengaku Zasshi* **26**, 243-248 (1981).

6. Enomoto, A. *et al.* Molecular identification of a renal urate anion exchanger that regulates blood urate levels.*Nature* **417**, 447-452 (2002).
7. Matsuo, H. *et al.* Mutations in glucose transporter 9 gene SLC2A9 cause renal hypouricemia. *Am. J. Hum. Genet.* **83**, 744-751 (2008).
8. Matsuo, H., Ichida, K., Takada, T., Nakayama, A. & Shinomiya, N. Urate transporters as controllers of urate dynamics. *Cell Technol*. **31**, 553-557 (2012) (in Japanese).
9. Kawamura, Y. *et al.* Pathogenic GLUT9 mutations causing renal hypouricemia type 2 (RHUC2). *Nucleosides Nucleotides Nucleic Acids* **30**, 1105-1111 (2011).
10. Matsuo, H. & Shinomiya, N. Genetics of renal hypouricemia. *Annual Rev. Diabetes/Metabolism/ Endocrinology* **2012**, 145-154 (2012) (in Japanese).
11. Ishikawa, I. Acute renal failure with severe loin pain and patchy renal ischemia after anaerobic exercise in patients with or without renal hypouricemia. *Nephron* **91**, 559-570 (2002).
12. Kikuchi, Y. *et al.* Patients with renal hypouricemia with exercise-induced acute renal failure and chronic renal dysfunction. *Clin. Nephrol.* **53**, 467-472 (2000).
13. Ichida, K. *et al.* Clinical and molecular analysis of patients with renal hypouricemia in Japan -Influence of URAT1 gene on urinary urate excretion. *J. Am. Soc. Nephrol.* **15**, 164-173 (2004).
14. Dinour, D. *et al.* Homozygous SLC2A9 mutations cause severe renal hypouricemia. *J. Am. Soc. Nephrol.* **21**, 64-72 (2010).
15. Sakiyama, M. *et al.* The effects of *URAT1/SLC22A12* nonfunctional variants, R90H and W258X, on serum uric acid levels and gout/hyperuricemia progression. *Sci. Rep.* **6**, 20148 (2016).

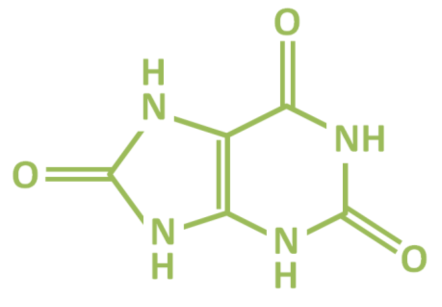

4 Guidance on the Diagnosis, Examination, and Differential Diagnosis of Renal Hypouricemia

Statement

1.	Hypouricemia is classified into two types: the overexcretion type and underproduction type, with the former being more common.	Consensus 5 ★★★★★
2.	Renal hypouricemia is a type of overexcretion-type hypouricemia. Hypouricemia (serum uric acid (S_{UA}) level of ≤ 2.0 mg/dl (120 μmol/l)) and increased fractional excretion of uric acid (FE_{UA}) and/or uric acid clearance (C_{UA}) by repeated tests are necessary for diagnosis of renal hypouricemia.	Consensus 5 ★★★★★
3.	Examination of FE_{UA} and/or C_{UA} should be performed using collected urine. FE_{UA} can, however, be calculated even with spot urine testing. Repeated tests of fasting FE_{UA} are also desirable.	Consensus 4 ★★★★☆
4.	Fanconi syndrome and xanthinuria are included as differential diagnoses of hypouricemia.	Consensus 5 ★★★★★

Summary

Renal hypouricemia (RHUC), which is classified as a type of overexcretion hypouricemia, is caused by increased urate excretion in the kidney despite the absence of tubular disorders[1-4]. It results from defective urate reabsorption transporters at the renal proximal tubules. Defects in the two urate reabsorption transporters URAT1/SLC22A12 and GLUT9/SLC2A9 are currently believed to be its causes[5-8]. RHUC itself is usually asymptomatic, but is reported to have complications, including exercise-induced acute kidney injury (EIAKI) and urolithiasis[6, 9-11]. Diagnosis of RHUC is important because paying attention to patients' lifestyle enables them to prevent these concomitant diseases.

1. Diagnostic guidance

It is essential for the diagnosis of RHUC to confirm increased urate-selective excretion in the kidney as well as to rule out other forms of hypouricemia. Acquisition of continuous findings by repeat testing are also necessary to diagnose RHUC[12]. The diagnostic guidance we have compiled in **Table 1** takes these facts into account. Note that due to the possibility of RHUC, repeated tests should be performed on patients whose serum uric acid (S_{UA}) level is 2.1 - 3.0 mg/dl (121 - 180 μmol/l), if they satisfy any of the following conditions:variants in causative genes of RHUC, past history of exercise-induced acute kidney

Table 1 Diagnostic guidance on renal hypouricemia (RHUC)

Required factors

Confirming continuous findings of **#1** and **#2**, while satisfying **#3**.	
**#1**	Hypouricemia with serum uric acid (S_{UA}) level of ≤ 2.0 mg/dl (120 μmol/l)*.
**#2**	Increased fractional excretion of uric acid (FE_{UA}) and/or uric acid clearance (C_{UA}).**
**#3**	Exclusion of other diseases that present hypouricemia (Table 4).

* There is a possibility of mild RHUC even with an S_{UA} of 2.1 - 3.0 mg/dl (121 - 180 μmol/l). Repeated tests of Required Factors **#1** and **#2** above are therefore desirable, especially when confirming any of the Reference Factors **1)** to **3)** shown below.

** The normal range of FE_{UA} and C_{UA} is 8.3 (5.5 - 11.1) % and 11.0 (7.3 - 14.7) ml/min, respectively.

Reference factors

1)	Mutations in the causative genes of RHUC (*URAT1/SLC22A12* and *GLUT9/SLC2A9* genes).
2)	Past history of exercise-induced acute kidney injury (EIAKI)***.
3)	Familial history of RHUC.

*** Because S_{UA} is not always lower during onset of EIAKI, S_{UA} should be checked before onset (if possible) or after amelioration.

injury (EIAKI), and family history of RHUC. Furthermore, there are studies that report the onset of EIAKI in patients whose S_{UA} was 2.1 - 3.0 mg/dl with increased renal urate excretion (mild RHUC)[13, 14]. Such patients, therefore, should be made aware of the risk of concomitant diseases.

2. Examination

Tests of S_{UA}, and fractional excretion of uric acid (FE_{UA}) and/or uric acid clearance (C_{UA}) are performed repeatedly for diagnosis of RHUC. Refer to Chapter 2 of the "Guideline for the management of hyperuricemia and gout (2nd edition)" for the details of these measuring methods and their normal range. Table 2 and Table 3 are excerpted from the Guideline[15] on the measuring method (60-minute method) of C_{UA}, creatinine clearance (C_{Cre}), fractional excretion of uric acid (FE_{UA}), and urinary urate excretion. The normal range of C_{UA} is 11.0 (7.3 - 14.7) mL/min, but is higher in RHUC cases. Because C_{UA} is often affected by other factors, such as urine volume, it is desirable to calculate FE_{UA} while simultaneously performing C_{Cre}. FE_{UA} can be calculated even with a spot urine test, and repeated

Table 2 Measuring method (60-minute method) of uric acid clearance and creatinine clearance

**3 days before test**	Limiting high-purine foods & alcohol intake
**Test day: after rising**	Fasting and drinking two cups of water
**Test day: at outpatient clinic**	–30 min: drinking 300 ml water<br>0 min: urination<br>30 min: midterm blood withdrawal [for S_{UA} and S_{Cre}]<br>60 min: urine collection [for urine volume, U_{UA}, U_{Cre}]

(Drawn from Reference 15)

Table 3 Calculation of uric acid clearance (C_{UA}), creatinine clearance (C_{Cre}), fractional excretion of uric acid (FE_{UA}), and urinary urate excretion (U_{UE})

$$C_{UA} = \frac{[\,U_{UA}\text{ (mg/dl)}\,] \times [\,\text{UV in 60 min (ml)}\,]}{[S_{UA}\text{ (mg/dl)}] \times 60} \times \frac{1.73}{BSA}$$

Normal range: 11.0 (7.3 to 14.7) ml/min

$$C_{Cre} = \frac{[\,U_{Cre}\text{ (mg/dl)}\,] \times [\,\text{UV in 60 min (ml)}\,]}{[S_{Cre}\text{ (mg/dl)}] \times 60} \times \frac{1.73}{BSA}$$

Normal range: 134 (97 to 170) ml/min

$$FE_{UA} = \frac{[C_{UA}]}{[C_{Cre}]} \times 100 = \frac{[U_{UA}] \times [S_{Cre}]}{[S_{UA}] \times [U_{Cre}]} \times 100$$

Normal range: 8.3 (5.5 to 11.1) %

*FE_{UA} is calculable even with spot urine, and repeated test of fasting FE_{UA} is desirable.

$$UUE = \frac{[U_{UA}\text{ (mg/dl)}] \times [\,\text{UV in 60 min (ml)}\,]}{100 \times [\,\text{Body weight (kg)}\,]}$$

Normal range: 0.496 (0.483 to 0.509) mg/kg/hr

UV: urine volume, BSA: body surface area

(Drawn from Reference 15)

testing of fasting FE_{UA} (normal range: 8.3 (5.5 - 11.1) %) is also desirable. The normal range of urinary urate excretion is 0.496 (0.483 - 0.509) mg/kg/hr., which is normal or often slightly higher in RHUC. To exclude other forms of overexcretion-type hypouricemia, examinations such as urinalysis (urinary specific gravity, pH, protein, sugar, and blood), urinary β2-microglobulin, and urinary N-acetyl-β-D-glucosaminidase (NAG) should be performed as necessary. Tests for diseases that cause hypouricemia should also be conducted as needed.

Table 4 Differential diagnosis of RHUC (Diseases which cause hypouricemia)

**1 Overexcretion-type hypouricemia**	
(1) Renal hypouricemia (RHUC)	(6) Diabetes mellitus
(2) Fanconi syndrome	(7) Drugs (such as benzbromarone and probenecid)
(3) Wilson's disease	(8) Pregnancy
(4) Syndrome of inappropriate secretion of antidiuretic hormone (SIADH)	(9) Intractable diarrhea
(5) Malignant tumor	

**2 Underproduction-type hypouricemia**	
(1) Xanthinuria (type I, type II)	(5) Idiopathic urate underproduction-type hypouricemia
(2) Molybdenum cofactor deficiency	(6) Severe hepatic injury
(3) Purine nucleoside phosphorylase deficiency (PNP deficiency)	(7) Drugs (such as allopurinol)
(4) PRPP synthetase hypoactivity	(8) Emaciation (malnutrition)

3. Differential diagnosis

Table 4 shows a list of diseases which show hypouricemia and which should be regarded as differential diagnoses of RHUC. Of these, excluding drugs, asymptomatic diseases showing only hypouricemia are RHUC and xanthinuria. Xanthinuria is distinguishable because xanthinuria shows little urinary urate excretion[16].

References

1. Praetorius, E., Kirk, J. E. Hypouricemia: with evidence for tubular elimination of uric acid. *J. Lab. Clin. Med.* **35**, 865-868 (1950).
2. Yeun, J. Y., Hasbargen, J. A. Renal hypouricemia: prevention of exercise-induced acute renal failure and a review of the literature. *Am. J. Kidney Dis.* **25**, 937-946 (1995).
3. Ishikawa, I. Acute renal failure with severe loin pain and patchy renal ischemia after anaerobic exercise in patients with or without renal hypouricemia. *Nephron* **91**, 559-570 (2002).
4. Sperling, O. Hereditary renal hypouricemia. *Mol. Genet. Metab.* **89**, 14-18 (2006).
5. Enomoto, A. *et al.* Molecular identification of a renal urate anion exchanger that regulates blood urate levels. *Nature* **417**, 447-452 (2002).
6. Ichida, K. *et al.* Clinical and molecular analysis of patients with renal hypouricemia in Japan-influence of URAT1 gene on urinary urate excretion. *J. Am. Soc. Nephrol.* **15**, 164-173 (2004).
7. Matsuo, H. *et al.* Mutations in glucose transporter 9 gene SLC2A9 cause renal hypouricemia. *Am. J. Hum. Genet.* **83**, 744- 751 (2008).
8. Dinour, D. *et al.* Homozygous SLC2A9 mutations cause severe renal hypouricemia. *J. Am. Soc. Nephrol.* **21**, 64-72 (2010).
9. Erley, C. M. *et al.* Acute renal failure due to uric acid nephropathy in a patient with renal hypouricemia. *Klin. Wochenschr.* **67**, 308- 312 (1989).
10. Ohta, T. *et al.* Exercise-induced acute renal failure associated with renal hypouricaemia: results of a questionnaire-based survey in Japan. *Nephrol. Dial. Transplant.* **19**, 1447- 1453 (2004).
11. Ishikawa, I. Acute renal failure. in *Renal hypouricemia*. p39-41 (Kanazawa Medical University Press, 2006) (in Japanese).
12. Hisatome, I. *et al.* Cause of persistent hypouricemia in outpatients. *Nephron* **51**, 13-16 (1989).
13. Ohtsuka, Y. *et al.* Human uric acid transporter 1 gene analysis in familial renal hypo-uricemia associated with exercise- induced acute renal failure. *Pediatr. Int.* **49**, 235-237 (2007).
14. Kaito, H. *et al.* Molecular background of urate transporter genes in patients with exercise-induced acute kidney injury. *Am. J. Nephrol.* **38**, 316-320 (2013).
15. The Guideline Revising Committee of the Japanese Society of Gout and Nucleic acid Metabolism. in *Guideline for the management of hyperuricemia and gout (2nd edition)*. p63-65 (Medical Review, 2010) (in Japanese).
16. Ichida, K. *et al.* Identification of two mutations in human xanthine dehydrogenase gene responsible for classical type I xanthinuria. *J. Clin. Invest.* **99**, 2391-2397 (1997).

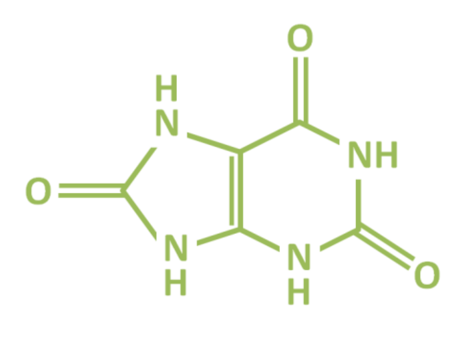

5 Concomitant Disease of Renal Hypouricemia (Exercise-Induced Acute Kidney Injury)

Statement

1.	Physicians should be aware that renal hypouricemia patients are at risk of exercise-induced acute kidney injury (EIAKI). Consensus 5 ★★★★★
2.	Differential diagnosis with myoglobinuric acute kidney injury which occurs after exercise is necessary. Consensus 4 ★★★★☆
3.	EIAKI cases generally show transient acute kidney injury (AKI), and receive common treatment for AKI. Consensus 4 ★★★★☆
4.	EIAKI carries a good short-term prognosis, although its long-term prognosis is unknown, and recurrence is also reported. Consensus 4 ★★★★☆
5.	For the prevention of EIAKI, some studies recommend drinking a large volume of water before exercise, limiting exercise while taking non-steroid anti-inflammatory drugs, and/or avoiding any strenuous exercise. Consensus 4 ★★★★☆
6.	There are some reports in which allopurinol, a xanthine oxidoreductase inhibiter, was administered as a preventive method based on a hypothetic mechanism of its pathogenesis; however, convincing evidence for its efficacy is lacking. Consensus 5 ★★★★★

Summary

Renal hypouricemia (RHUC) patients often suffer from one of its concomitant diseases, exercise-induced acute kidney injury (EIAKI), that is, acute kidney injury (AKI) with loin pain and without findings of increased serum creatine kinase and myoglobin after strenuous (anaerobic) exercise. Although its mechanism of pathogenesis remains to be clarified, recent diagnostic imaging suggests a relationship with renal ischemia.

Almost all cases show transient AKI, but there are no reports of RHUC patients eventually requiring long-term dialysis. However, there are reports of cases who required short-term dialysis therapy due to oliguria, uremia, electrolyte abnormalities, or heart failure, and other case reports of its concomitance with posterior reversible encephalopathy syndrome (PRES), a syndrome showing acute brain edema with neurological symptoms. There is no evidence on the long-term prognosis of EIAKI.

The main treatment for EIAKI is the same as that for common AKI. Some tentative preventive methods have been reported, but the evidence for their effectiveness is not conclusive.

1. Diagnosis and characteristics of exercise-induced acute kidney injury (EIAKI)

Renal hypouricemia (RHUC) patients are often reported to have one of its concomitant diseases, exercise-induced acute kidney injury (EIAKI), that is, acute kidney injury (AKI) with loin pain and without (or with slight) findings of increased creatine kinase (CK) and myoglobin after relatively brief but strenuous (anaerobic) exercise. EIAKI is thought to have a different pathogenesis from that of AKI, with myoglobinuria caused by exercise-induced rhabdomyolysis. Because EIAKI often causes severe loin pain, EIAKI is also termed "acute kidney injury with severe loin pain and patchy renal ischemia after anaerobic exercise" (ALPE)[1].

Unlike myoglobinuric AKI, EIAKI is often caused by strenuous anaerobic exercise such as short-distance sprinting, soccer, muscle-building exercise, cycling, and weight-lifting. Its patients are also often observed to have taken anti-inflammatory analgesics for cold symptoms[2]. The median age of onset is 19, and the male-female ratio is 202:18, making EIAKI a predominantly male disease[2]. Urolithiasis-like subjective symptoms such as loin pain and nausea/vomiting occur 1 - 48 hours after exercise. Compared to myoglobinuric AKI, EIAKI shows milder dehydration and is a non-oliguric form of AKI.[3] EIAKI is also observed without rust-colored urine (albuminuria), and with slightly increased serum CK and myoglobin: serum CK and myoglobin is up to nine times and seven times the upper normal range, respectively[1]. EIAKI normally has an average disease duration of 14 days.[3]

2. Are RHUC patients more susceptible to EIAKI than the normouricemic population?

From a study of 201 cases of EIAKI, the number of RHUC patients came to 116 (57.7%)[2]. Since the prevalence of RHUC is estimated to be 0.2% - 0.4% (see Chapter 2), it can be assumed that RHUC patients are more susceptible to EIAKI than the normouricemic population. If RHUC cases found after onset of EIAKI are excluded, the frequency of EIAKI with RHUC was observed to be two of 31 cases (6.5%)[4]. Meanwhile, 15 of 71 RHUC cases (21.1%)[5] and 13 of 54 patients (24.1%)[6] were found to have past histories of EIAKI in studies in which RHUC cases diagnosed after onset of EIAKI were included. In future studies, the prevalence of EIAKI among RHUC patients, including mild cases, will need to be known through epidemiological investigations.

According to the above-mentioned study, 43 of 221 cases (19.5%) experienced recurrence[1]. Furthermore, EIAKI is reported to be accompanied by a syndrome showing acute brain edema with neurological symptoms, termed posterior reversible encephalopathy syndrome (PRES)[7,8].

For the above reasons, all RHUC patients should be made aware of the risk of onset and recurrence of EIAKI.

3. Pathogenesis of EIAKI on RHUC cases

The pathogenesis of EIAKI in RHUC patients is not known, but a variety of hypotheses have been proposed. One of these advocates that lowered serum uric acid level in RHUC patients results in a

lowered radical scavenger effect of urate, which aggravates the ischemic effect activated by renal vasopressor factors due to oxygen radicals and unknown substances produced during anaerobic exercise. Based on this hypothesis, some studies have revealed the presence of an oxidant imbalance using interventional trials of exercise stress in EIAKI cases without hypouricemia.[9,10] Other studies have shown a reversible resistance index using ultrasonography of renal blood flow in EIAKI patients[11]. There is also a study[2] that reports, using delayed CT, a wedge-shaped residue of contrast agent in the kidney lasting a few hours to several days after injection of 40 ml contrast agent during the return period of kidney function (serum creatinine of 1.5 - 3.0 mg/dl), whereas myoglobinuric AKI displays a diffuse residue. Conducting contrast-enhanced CT in AKI patients, of course, needs particular attention. There is a study[12] in which diffusion-weighted MRI imaging displayed wedge-shaped ischemic lesions.

There is another hypothesis that proposes obstructive renal injury caused by rapid crystallization of urate onto the renal tubules; however, no renal biopsies have been found that provide evidence of this hypothesis.

4. Treatment of RHUC patients with EIAKI

Some case studies report that patients had received conventional treatment of AKI to increase renal blood flow, such as appropriate rehydration therapy and administration of calcium blockers and dopamine preparations. There are also various individuals who were administered allopurinol for the reasons described below. Hemodialysis would be indicated if they experienced oliguria, uremia, electrolyte abnormalities, or heart failure, but there are no studies that report a move to long-term dialysis due to exacerbation to chronic kidney disease (CKD). A review by Ishikawa (34 of 116 cases)[2] and other case studies report that some individuals required short-term dialysis.

5. Prevention of EIAKI in RHUC patients

EIAKI onset often occurs in RHUC patients, but its unpredictability disrupts interventional trials in a certain number of cases, which results in few studies with sufficient evidence to draw conclusions. Some case studies report the following findings.

There is a hypothesis that EIAKI in RHUC patients is caused by the renal vasopressive effects of oxygen radicals, which are produced by xanthine oxidase (XO). XO is the oxidase form of xanthine oxidoreductase (XOR) which synthesizes urate. Based on this hypothesis, some studies recommend the administration of XOR inhibitors to RHUC patients. In one report, healthy controls, including athletes dosed with allopurinol (an XOR inhibitor) before exercise, showed lower serum values of enzymes released from dead cells and myoglobin levels[13]. Other reports showed that administering allopurinol to RHUC patients before exercise decreased urinary urate excretion and might thus prevent EIAKI[14,15]. Although these effects were assumed to be caused by allopurinol, which reduces radical oxygen by XO and/or decreases urinary urate excretion, these reports are case reports and do not carry sufficient evidence to

permit recommendations to be included in clinical practice guidelines. Administering XOR inhibitors, however, has the potential to prevent the onset and/or recurrence of EIAKI. Therefore, application should be decided with careful consideration of its benefits and harms, especially for those with a risk of EIAKI, such as athletes and/or individuals with a past history of the conditions.

Furthermore, to prevent EIAKI, there is a study[2] that recommends patients to drink water before exercise, not to take non-steroid anti-inflammatory drugs (NSAIDs), or to avoid exercise while taking NSAIDs, and/or to avoid all strenuous exercise.

References

1. Ishikawa, I. Uric acid increase after exercise and exercise-induced acute renal failure. *J. Adult Dis.* **43**, 970-975 (2013) (in Japanese).
2. Ishikawa, I. ALPE. *Gout Nucleic Acid Metab*. **34**, 145-157 (2010) (in Japanese).
3. Ishikawa, I. Acute renal failure induced by exercise. *J. Jpn. Soc. Int. Med.* **99**, 970-976 (2010) (in Japanese).
4. Ichida, K. *et al.* Clinical and molecular analysis of patients with renal hypouricemia in Japan-influence of URAT1 gene on urinary urate excretion. *J. Am. Soc. Nephrol.* **15**, 164-173 (2004).
5. Ichida, K. *et al.* Age and origin of the G774A mutation in SLC22A12 causing renal hypouricemia in Japanese. *Clin. Genet.* **74**, 243-251 (2008).
6. Ohta, T. *et al.* Exercise-induced acute renal failure associated with renal hypouricaemia: results of a questionnaire-based survey in Japan. *Nephrol. Dial. Transplant.* **19**, 1447-1453 (2004).
7. Shima, Y. *et al.* A case report of renal hypouricemia with recurrent EIARF and PRES. *J. Jpn. Soc. Pediatr. Ren. Fail.* **30**, 145-146 (2010) (in Japanese).
8. Shima, Y. *et al.* Recurrent EIARF and PRES with severe renal hypouricemia by compound heterozygous SLC2A9 mutation. *Pediatrics* **127**, e1621-1625 (2011).
9. Kaneko, K. *et al.* Oxidative imbalance in idiopathic renal hypouricemia. *Pediatr. Nephrol.* **24**, 869-871 (2009).
10. Karasawa, T. *et al.* A case report of exercise-induced acute renal failure. *Jpn. J. Pediatr. Nephrol.* **23**, 96-101 (2010) (in Japanese).
11. Saito, O. *et al.* Two cases of renal hypouricemia in which dopamine infusion produced a good recovery from exercise-induced acute kidney injury. *Clin. Nephrol.* **76**, 83-90 (2011).
12. Ohta, K. *et al.* Diffusion-weighted MRI of exercise-induced acute renal failure (ALPE). *Pediatr. Nephrol.* **26**, 1321-1324 (2011).
13. Sanchis-Gomar, F. *et al.* Effects of allopurinol on exercise-induced muscle damage: new therapeutic approaches? *Cell Stress Chaperones* **20**, 3-13 (2015).
14. Bhasin, B. *et al.* Hereditary renal hypouricemia: a new role for allopurinol? *Am. J. Med.* **127**, e3-4 (2014).
15. Yeun, J. Y., Hasbargen, J. A. Renal hypouricemia: prevention of exercise-induced acute renal failure and a review of the literature. *Am. J. Kidney Dis.* **25**, 937-946 (1995).

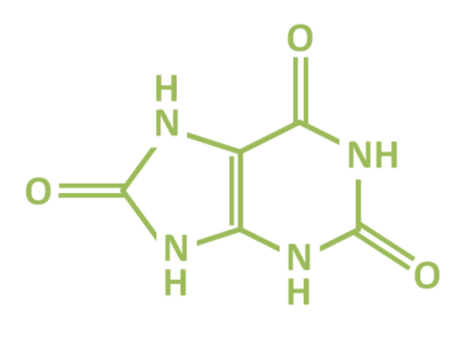

6 Concomitant Disease of Renal Hypouricemia (Urolithiasis)

Statement

1.	Renal hypouricemia patients should be aware of the risk of developing kidney stones. Consensus 5 ★★★★★
2.	If uric acid stones develop, dissolution therapy by urinary alkalization is also effective. Consensus 4 ★★★★☆
3.	To prevent urolithiasis, fluid intake that will achieve a urine volume of at least 2,000 ml daily is recommended. Consensus 4 ★★★★☆
4.	Citrate compounds can be prescribed to renal hypouricemia patients with urinary stones to maintain urinary pH at an optimal level of 6.0 - 7.0 to prevent stone occurrence. Consensus 4 ★★★★☆

Summary

Renal hypouricemia (RHUC) often causes urinary stones, such as uric acid or calcium oxalate stones. Ultrasonotomography and computed tomography are used as diagnostic imaging tools. Plain film of the kidneys, ureter, and bladder (KUB) is not useful, because uric acid stones are radiolucent. Urinalysis, including urine pH, crystal in urinary sediment, and urinary urate excretion during 24-hour urine collection, is useful for diagnosis. Analysis of delivered stones is also desirable to know which kind of stones they are.

Extracorporeal shock wave lithotripsy (ESWL), percutaneous nephro-ureterolithotripsy (PNL), and transurethral ureterolithotripsy (TUL) are aggressive treatments for removing these stones. Dissolution therapy of uric acid stones by urinary alkalinization is also effective. It is mandatory for fluid intake that will achieve a urine volume of at least 2,000 ml daily to prevent urinary stones. Urine alkalinization using citrate compounds is necessary to maintain urinary pH between 6.0 and 7.0.

1. Introduction

Urolithiasis a common concomitant disease of RHUC. This is because increased urate concentrations in patients' renal tubules result in increased urinary urate excretion. While urinary stones in Japanese RHUC1 patients, that is, RHUC due to variants in the *URAT1/SLC22A12* gene, reportedly occur in 6.1 - 8.5%,[1,2] actual numbers of patients are likely to be greater if asymptomatic urolithiasis is taken into account.

More common development of urinary stones is predicted for RHUC2 patients (RHUC with variants in the *GLUT9/ SLC2A9* gene) than in RHUC1[3], because of the higher urate concentration in the renal tubules.

Not only uric acid stones, but also calcium oxalate stones (pure calcium oxalate, or mixed with uric acid) are observed as urinary stones in RHUC cases[4]. This finding accords with the characteristics of urinary stones that are concomitant with hyperuricemia and gout[5].

Urate crystal deposition in the renal tubules and/or collecting ducts, and crystal adherence onto the urothelium are thought to be the chief mechanism of development of uric acid stones[6]. In the case of calcium oxalate stones, heterogeneous nucleation, in which a calcium oxalate stone grows after a uric acid stone has formed as its nucleus[7], and increased crystallization of calcium oxalate stone due to the salting-out effect[8] have been suggested. It is further hypothesized that tubular obstruction by uric acid crystals also affects the onset of exercise-induced acute kidney injury (EIAKI)[9,10] and the formation of calcium oxalate stones[11].

If the patient is diagnosed with RHUC as well as EIAKI, long-term observation and the adoption of preventive measures against urinary stones are necessary.

2. Diagnosis

In the same manner as for other urinary stones, ultrasonotomography is used for diagnostic imaging of urinary stones seen with RHUC[12]. Ultrasonotomography can be used to detect stones in the kidney, upper ureter, and nearby bladder, although it is less effective in detecting stones in other regions. It is also a problem that, although easy to perform, the results of ultrasonotomography depend closely on the skills of the practitioner. Plain X-ray film of the abdomen (also known as plain kidney, ureter, and bladder radiography, or KUB) can be used to observe calcium-containing stones, whereas it is incompatible with radiolucent stones, such as uric acid stones. Computed tomography (CT) is the most useful tool for gaining a definite diagnosis of all urinary stones associated with urate metabolism[13], making it the first choice, especially in an emergency room situation. The latest dual-energy CT scanners, which use several X-ray sources, make it possible not only to diagnose stones, but also to conduct an analysis of the stone's components:[14] stone types are mostly distinguishable using this form of CT imaging only because uric acid stones have similar CT numbers between high- and low-voltage acquisitions, while calcium-containing stones have distinct numbers.

It is essential in urinalysis to observe urinary pH and crystalline components in urinary sediment. Yellowish-brown, irregular plate-like crystals in aciduria (pH < 6.0) indicate the presence of uric acid stones.

Evaluating urinary urate excretion by chemical examination of 24-hour urine collection is preferable for detecting urinary stones, whereas calculating FE_{UA} from fasting spot urine allows RHUC to be readily diagnosed. The Japanese clinical guideline for the treatment of urolithiasis (first edition) defines the normal range of urinary

urate excretion in 24-hour urine collection as < 800 mg/day for males and < 750 mg/day for females[15]. Hyperuricosuria is therefore diagnosed when these ranges are exceeded.

It is desirable to perform calculus analysis of excreted stones as soon as possible, because knowledge of the stone's composition enables treatment to prevent recurrence. Analysis of stone components is mainly conducted by Fourier transform infrared spectrophotometer, since it is a low-cost, high-sensitivity, high-accuracy, and easy for data processing method. In infrared spectroscopic analysis, stone components are identified from their infrared absorption spectrum, which is obtained from the dispersed transmitted light of powdered stone when irradiated with infrared light[16].

3. Treatment

The treatment strategy for urinary stones concomitant with RHUC is the same as for other stones. If the stones are not delivered spontaneously, or they are causing symptoms such as prolonged pain, inflammatory findings, or loss of renal function, the subject should be treated using stone removal therapy. Endourological treatments such as extracorporeal shock wave lithotripsy (ESWL), percutaneous nephro-ureterolithotripsy (PNL), or transurethral ureterolithotripsy (TUL) are performed as aggressive treatments. Their application will differ according to the region and the size of the stones[17]. If uric acid stones are suspected using the imaging diagnosis described above, dissolution therapy by administering urinary alkalinizers is also likely to be effective, even in patients with RHUC[18]. However, it will take a long time for the stones to dissolve completely.

4. Prevention of stone recurrence

The risk factors for urinary stones that are concomitant with hyperuricemia and gout are (1) low urinary volume or poor intake of water, (2) increased urinary urate excretion, (3) and presence of aciduria[5]. These are the same as for urinary stones caused by RHUC.

The aim of the advice to drink plenty of water is to decrease urinary urate concentration and saturation. Its goal is to secure a urinary volume of more than 2,000 ml/day. Patients should be advised to avoid alcohol and beverages containing high levels of sugar and/or purine bodies as their source of water. There are not enough reports to be able to evaluate the effect of diet treatment on RHUC cases. However, to be able to remove the risk factors for urinary stones described above, consuming alkaline foods such as vegetables and seaweed is desirable. RHUC patients should also avoid consuming large amounts of purine-containing foods, since these can cause an increase in urinary urate excretion. Conversely, conscientiously limiting purine bodies diligently would be less useful.

The solubility of uric acid in urine is reported to be 15 mg/dl when urinary pH is 5.0, whereas it is 200 mg/dl at pH 7.0. Urinary alkalinization is necessary to treat aciduria in RHUC cases, because the higher the urinary pH, the greater the solubility of uric acid. The concomitance of RHUC and aciduria is also reported[19]. Citrate

compounds (potassium citrate and sodium citrate) are mainly used as a urinary alkalinizers. They should be administered to maintain patients' urinary pH at ≥ 6.0 and < 7.0, because excess urinary alkalinization (urinary pH ≥ 7.5) promotes the deposition of calcium phosphate and sodium urate.

Although both urinary alkalinization and administration of xanthine oxidoreductase (XOR) inhibitors are generally recommended[20] to prevent the recurrence of uric acid stones and calcium oxalate stones, few studies report the effects of XOR inhibitors on urinary stones in RHUC patients. One study reports that XOR inhibitors such as allopurinol, febuxostat, and topiroxostat decrease the filtered uric acid load and reduce its crystallization in the renal tubules, resulting in decreased EIAKI onset[21]. Combined usage of XOR inhibitors and urinary alkalinizers might therefore be considered when patients' urinary urate excretion has increased.

References

1. Ichida, K. *et al.* Age and origin of the G774A mutation in SLC22A12 causing renal hypouricemia in Japanese. *Clin. Genet.* **74**, 243-251 (2008).
2. Shinomiya, N. Studies to Reveal the Incidence of Renal Hypouricemia Nationwide. *Final report for a Health and Labour Sciences Research Grant* (2014) (in Japanese).
3. Dinour, D. *et al.* Homozygous SLC2A9 mutations cause severe renal hypouricemia. *J. Am. Soc. Nephrol.* **21**, 64-72 (2010).
4. Sugishita, N., Ishikawa, I. & Tateichi, K. A case of hereditary renal hypouricemia with uric acid renal stones. *Kid. Dial.* **15**, 277-281 (1983) (in Japanese).
5. The Guideline Revising Committee of the Japanese Society of Gout and Nucleic acid Metabolism. in *Guideline for the management of hyperuricemia and gout (2nd edition)*. p44-45 (Medical Review, 2010) (in Japanese).
6. Riese, R. J., Kleinman, J. G., Wiessner, J. H., Mandel, G. S. & Mandel, N. S. Uric acid crystal binding to renal inner medullary collecting duct cells in primary culture. *J. Am. Soc. Nephrol.* **1**, 187-192 (1990).
7. Meyer, J. L., Bergert, J. H. & Smith, L. H. The epitaxially induced crystal growth of calcium oxalate by crystalline uric acid. *Invest. Urol.* **14**, 115-119 (1976).
8. Grover, P. K., Marshall, V. R. & Ryall, R. L. Dissolved urate salts out calcium oxalate in undiluted human urine in vitro: implications for calcium oxalate stone genesis. *Chem. Biol.* **10**, 271-278 (2003).
9. Jeannin, G. *et al.* Recurrent exercise-induced acute renal failure in a young Pakistani man with severe renal hypouricemia and SLC2A9 compound heterozygosity. *BMC Med. Genet.* **15**, 3 (2014).
10. Erley, C. M., Hirschberg, R. R., Hoefer, W. & Schaefer, K. Acute renal failure due to uric acid nephropathy in a patient with renal hypouricemia. *Klin. Wochenschr.* **67**, 308-312 (1989).
11. Coe, F. L. Uric acid and calcium oxalate nephrolithiasis. *Kidney Int.* **24**, 392-403 (1983).
12. Japanese Urological Association, Japanese Society of Endourology & Japanese Society on urolithiasis research. in *Japanese urolithiasis clinical guideline (2nd edition)* p37-39 (Kanehara & Co., Ltd., 2013) (in Japanese).
13. Shimizu, T., Kitada, H., Umeyama, M., Hori, H. & Takasaki, N. Novel evaluation of nephrolithiasis as a complication of gout: a cross-sectional study using helical computerized tomography. *J. Urol.* **189**, 1747-1752 (2013).
14. Yamaguchi, S. *et al.* Application of dual energy CT for qualitative diagnosis on stone constituent and its clinical limitation. *Jpn. Soc. Urolithiasis Res.* **12**, 96-100 (2014) (in Japanese).
15. Japanese Urological Association, Japanese Society of Endourology and ESWL, & Japanese Society on urolithiasis research. in *Japanese urolithiasis clinical guideline (2nd edition)* p59-61 (Kanehara & Co., Ltd., 2002) (in Japanese).
16. Tozuka, K. in *All of Urolithiasis: a Complete Guide* (ed. Japanese Society of Urolithiasis Research) p41-42 (Igaku Shoin, 2008) (in Japanese).
17. Japanese Urological Association, Japanese Society of Endourology & Japanese Society on urolithiasis research. In *Japanese urolithiasis clinical guideline (2nd edition)* p30-36 (Kanehara & Co., Ltd., 2013) (in Japanese).
18. Hisatome, I. *et al.* Excess urate excretion correlates with severely acidic urine in patients with renal hypouricemia. *Intern. Med.* **37**, 726-731 (1998).
19. Hisatome, I. *et al.* Renal hypouricemia due to enhanced tubular secretion of urate associated with urolithiasis: successful treatment of urolithiasis by alkalization of urine K+, Na+-citrate. *Nephron* **65**, 578-582 (1993).
20. Klinenberg, J. R., Goldfinger, S. E. & Seegmiller, J. E. The effectiveness of the xanthine oxidase inhibitor allopurinol in the treatment of gout. Ann. *Intern. Med.* **62**, 639-647 (1965).
21. Bhasin, B. *et al.* Hereditary renal hypouricemia: a new role for allopurinol? *Am. J. Med.* **127**, e3-4 (2014).

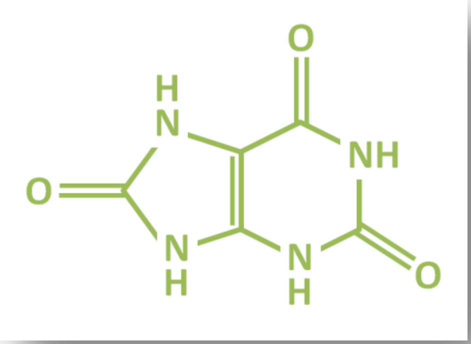
O
H
N
NH
O
N
H
N
H
O

7 Clinical Questions and Recommendations

CQ1 Should individuals with a serum uric acid level of ≤ 2.0 mg/dl be considered for differential diagnosis of hypouricemia?

Recommendation 1 We strongly recommend that individuals who have a serum uric acid level of ≤ 2.0 mg/dl (120 μmol/l) be considered for differential diagnosis of hypouricemia.

Expository comment

Hypouricemia is often a finding discovered by chance during ordinary medical examinations. Low serum uric acid (S_{UA}) level is one of the characteristics of renal hypouricemia (RHUC), although there has been no clear cutoff threshold of S_{UA} to officially suspect RHUC.

Table 1 Representative reports on the epidemiological distribution of serum uric acid (S_{UA}) level.

Report	Information source	Percentage of individuals with S_{UA} ≤ 2.0 mg/dl (120 μmol/l) (Total tested population)		Percentage of individuals with S_{UA} ≤ 3.0 mg/dl (180 μmol/l) (Total tested population)	
		Male	Female	Male	Female
Sasaki (1980) [1]	Medical examination (ME)	1.07% (13,909)	10.05% (4,785)	-	-
Gresseer (1990) [2]	Blood donors	0.0% (2,097)	0.3% (1,103)	0.3% (2,097)	8.3% (1,103)
Kaneko (1995) [3]	Comprehensive ME	0.11% (55,735)	0.44% (35,555)	-	-
Tabe (1996) [4]	Comprehensive ME and ME	0.14% (17,603)	0.40% (3,544)	0.48% (16,071)	3.87% (3,335)
Hamajima (2011) [5]	ME[Note 1]	0.0% (3,256)	0.1% (1,537)	0.1% (3,256)	3.5% (1,537)
Matsuo (2008) [6]	ME[Note 2]	0.18% (21,260)		0.94% (21,260)	

Note 1) The tested population is that without the W258X mutation: a dysfunctional mutation of the urate reabsorption transporter gene *URAT1/SLC22A12* which causes renal hypouricemia type 1. Therefore, most RHUC cases should be excluded from this population. (RHUC cases not caused by W258X are, however, included.)
Note 2) Most of the tested population is male, since they were recruited from Self-Defense Forces (military) personnel.

Table 1 shows typical reports obtained through a systematic review of the literature which examined the epidemiological distribution of S_{UA}, which suggest that there are considerable numbers of people, especially female, with an S_{UA} of 2.0 - 3.0 mg/dl (120 - 180 μmol/l) that do not show RHUC. Otherwise, this population seems to be adequately excluded with a cutoff value of S_{UA} set at ≤ 2.0 mg/dl.

Moreover, few RHUC cases with concomitant diseases (exercise-induced acute kidney injury [EIAKI] and urolithiasis) had a normal S_{UA} of > 2.0 mg/dl[7-10].

However, re-examination of S_{UA} sometimes shows a low S_{UA} of ≤ 2.0 mg/dl even they initially showed S_{UA} of > 2.0 mg/dl[11,12]. Furthermore, there is a study reporting that heterozygous carriers of the *URAT1/SLC22A12* gene show various values of S_{UA} (often > 3.0 mg/dl)[5]. Therefore, because individuals with variants in their urate transporter genes often show an S_{UA} of > 2.0 mg/dl[5–9,13,14], re-examining S_{UA} several times and searching for genetic variants (if necessary) would be helpful, especially if they have a past history of EIAKI and/or urolithiasis, or a family history of hypouricemia.

When their S_{UA} values are ≤ 1.0 mg/dl, RHUC and xanthinuria should be considered as a differential diagnosis. Xanthinuria, however, is easily distinguished because its disease frequency is much lower than that of RHUC, and because xanthinuria results in little urinary urate excretion and little fractional excretion of uric acid. Syndrome of inappropriate secretion of antidiuretic hormone (SIADH) and diabetes mellitus are known to be causes of secondary hypouricemia. However, they are relatively distinguishable from RHUC because they often show S_{UA} of > 2.0 mg/dl and other findings such as hyponatremia and hyperglycemia[12,15]. Likewise, secondary hypouricemia caused by Wilson's disease or Fanconi syndrome, respectively, is also relatively identifiable due to the presence of other clinical evidence, including hypoceruloplasminemia or hyperaminoaciduria/hyperphosphatemia. Hypouricemia caused by drugs or tumors is distinguishable from RHUC by its clinical course.

References

1. Sasaki, S. Congenital hypouricemia. *Ryumachi. [Rheumatism]* **20**, 95-106 (1980).
2. Gresser, U., Gathof, B. & Zollner, N. Uric acid levels in southern Germany in 1989. A comparison with studies from 1962, 1971, and 1984. *Klin. Wochenschr*. **68**, 1222-1228 (1990).
3. Kaneko, K. *et al*. Hypouricemia: frequency and clinical significance of low uric acid level. *Medical Practice* **12**, 659-662 (1995) (in Japanese).
4. Tabe, A. Research on the pathophysiology of hypouricemia. *Tokyo Jikei-kai Ika Daigaku Zasshi* **111**, 821- 839 (1996) (in Japanese).
5. Hamajima, N. *et al*. Serum uric acid distribution according to SLC22A12 W258X genotype in a cross-sectional study of a general Japanese population. *BMC Med. Genet*. **12**, 33 (2011).
6. Matsuo, H. *et al.* Mutations in glucose transporter 9 gene SLC2A9 cause renal hypouricemia. *Am. J. Hum. Genet.* **83**, 744-751 (2008).
7. Ichida, K. *et al.* Age and origin of the G774A mutation in SLC22A12 causing renal hypouricemia in Japanese. *Clin. Genet*. **74**, 243-251 (2008).
8. Dinour, D. *et al*. URAT1 mutations cause renal hypouricemia type 1 in Iraqi Jews. *Nephrol. Dial. Transplant*. **26**, 2175-2181 (2011).
9. Jeannin, G. *et al*. Recurrent exercise-induced acute renal failure in a young Pakistani man with severe renal hypouricemia and SLC2A9 compound heterozygosity. *BMC Med. Genet*. **15**, 3 (2014).

9. Ohta, T. *et al*. Exercise-induced acute renal failure associated with renal hypouricaemia: results of a questionnaire-based survey in Japan. *Nephrol. Dial. Transplant*. **19**, 1447-1453 (2004).
10. Smetana, S. S. & Bar-Khayim, Y. Hypouricemia due to renal tubular defect. A study with the probenecid-pyrazinamide test. *Arch. Int. Med*. **145**, 1200-1203 (1985).
11. Shichiri, M., Itoh, H., Iwamoto, H., Hirata, Y. & Marumo, F. Renal tubular hypouricemia: evidence for defect of both secretion and reabsorption. *Nephron* **56**, 421-426 (1990).
12. Dinour, D. *et al*. Homozygous SLC2A9 mutations cause severe renal hypouricemia. *J. Am. Soc. Nephrol*. **21**, 64-72 (2010).
13. Dinour, D. *et al*. Two novel homozygous SLC2A9 mutations cause renal hypouricemia type 2. *Nephrol. Dial. Transplant*. **27**, 1035-1041 (2012).
14. Beck, L. H. Hypouricemia in the syndrome of inappropriate secretion of antidiuretic hormone. *N. Engl. J. Med*. **301**, 528-530 (1979).

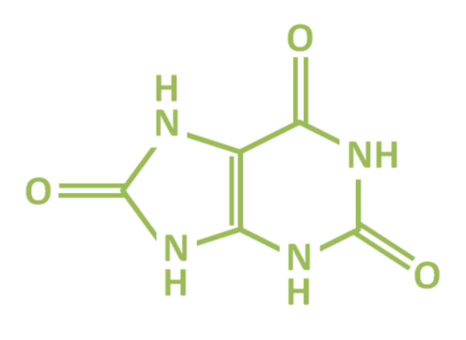

CQ2 Should xanthine oxidoreductase (XOR) inhibitors be administered to prevent exercise-induced acute kidney injury (EIAKI) in patients with renal hypouricemia (RHUC)?

Recommendation 2 We cannot definitively state that XOR inhibitors should be administered to prevent EIAKI in RHUC patients. However, it is possible that XOR inhibitors might prevent the onset or relapse of EIAKI. Administration of XOR inhibitors should therefore be decided in the light of its potential benefits and harms, especially for athletes and high-risk patients with a past history of EIAKI attacks.

Expository comment

Patients with renal hypouricemia (RHUC) often suffer from recurrence of its concomitant diseases, including exercise-induced acute kidney injury (EIAKI) and urolithiasis. To prevent this, patients are usually recommended to drink a lot (especially before exercise), to avoid strenuous exercise, and/or to avoid all exercise when taking non-steroid anti-inflammatory drugs, sometimes combined with the administration of urinary alkalinizers to prevent urolithiasis. The administration of xanthine oxidoreductase (XOR) inhibiters (as of 2015, allopurinol, febuxostat, and topiroxostat are available in Japan) before exercise is also assumed to prevent EIAKI. The clinical effectiveness of XOR inhibitors, however, remains unclear and needs to be investigated.

Since urate has a strong antioxidant effect, it might mitigate vasopressor effects in the kidney caused by oxygen radicals produced during anaerobic exercise. This has prompted a hypothesis to explain why EIAKI is concomitant with RHUC: namely, that low serum uric acid levels in RHUC patients lead to lower antioxidant effects (the scavenger effect), which results in prolonged renal ischemia due to continuous vasopressor effects in the kidney. Some, but not much, of the literature provides evidence for this hypothesis, as listed below.

- The renal blood flow of two RHUC patients was examined using Doppler ultrasonography: they showed a reversible resistance index which indicates transient instability of renal hemodynamics[1].
- Contrast-enhanced CT was conducted for RHUC with EIAKI. It revealed a wedge-shaped residue of contrast agent in the kidney, lasting from several hours to several days after injection. This finding indicates the presence of renal ischemia[2].
- Although hypouricemia patients were not the main target of the study, interventional trials of exercise stress were performed in EIAKI patients: d-ROMs and BAP tests[3] revealed the presence of oxidant imbalance.

Xanthine oxidase (XO), the oxidase form of XOR, produces oxygen radicals as well as urate along the urate synthesis metabolic pathway. While XOR inhibitors are usually used as urate-lowering therapy to act as urate-synthesis inhibitors, they also inhibit the production of oxygen radicals. It is therefore possible that XOR inhibitors mitigate the deterioration of renal hemodynamics in RHUC patients.

There are a few studies that provide evidence of the usefulness of administering XOR inhibitors to RHUC patients to prevent EIAKI. All of these are case reports.

- Exercise stress tests were performed with Pakistani RHUC patients, and renal dysfunction was induced, but it could be prevented by premedication with allopurinol (300 mg/day for five days). Four healthy controls did not show renal dysfunction in the exercise stress tests[4].
- In a report from the USA, an RHUC patient, a 400 meter-sprinter, had experienced EIAKI, but after recovering, EIAKI did not recur following track meetings after premedication with allopurinol (300 mg/day for three days)[5].
- Healthy controls including athletes prescribed with allopurinol before exercise showed decreased serum values of enzymes released from dead cells (such as creatinine kinase) and myoglobin levels, indicating reduced damage to skeletal muscle[6].

As described above, there are a few reports that suggest the potential for the use of allopurinol to prevent EIAKI in RHUC patients. There are, however, no reports featuring Japanese/East Asians, randomized intervention studies, or studies with multiple patients. Moreover, no studies were found in which the amount and duration of allopurinol were evidenced.

It is not appropriate to administer XOR inhibitors to all RHUC patients to prevent EIAKI. This is because not all RHUC patients suffer from EIAKI, and because administration of XOR inhibitors could cause adverse events. Administering XOR inhibitors, however, may provide significant benefits, especially to those at risk of EIAKI, such as individuals with a past history of EIAKI and/or athletes, taking into account the fact that common and relatively low-cost drugs are reported, in a few studies, to prevent the serious concomitant diseases of EIAKI.

We cannot find any evidence on whether the risk of adverse effects from XOR inhibitors is higher in RHUC patients than in patients with other diseases treated with XOR inhibitors, such as hyperuricemia.

In response to the discussion above, to the clinical question (CQ) “Should xanthine oxidoreductase (XOR) inhibitors be administered to prevent exercise-induced acute kidney injury (EIAKI) in patients with renal hypouricemia (RHUC)?,” our conclusion is “We cannot definitively state that XOR inhibitors should be administered to prevent EIAKI in RHUC patients.” We also present our recommendation that “Administration of XOR inhibitors should be decided by taking into account both benefits and harms, especially for patients known to be at high risk, such as athletes or individuals with

a past history of EIAKI,” because it is possible that XOR inhibitors might prevent the onset or relapse of EIAKI. Future research is needed on this key clinical issue.

References

1. Saito, O. *et al.* Two cases of renal hypouricemia in which dopamine infusion produced a good recovery from exercise-induced acute kidney injury. *Clin. Nephrol.* **76**, 83-90 (2011).
2. Yamada, A. Exercise-indeced acute kidney injury. *Kyukyu Shuchu Chiryo* **24**, 444-447 (2012). (in Japanese)
3. Karasawa, T. *et al*. A case report of exercise-induced acute renal failure. *Jpn. J. Pediatr. Nephrol*. **23**, 96-101 (2010). (in Japanese)
4. Yeun, J. Y. & Hasbargen, J. A. Renal hypouricemia: prevention of exercise-induced acute renal failure and a review of the literature. *Am. J. Kidney Dis.* **25**, 937-946 (1995).
5. Bhasin, B. *et al*. Hereditary renal hypouricemia: a new role for allopurinol? *Am. J. Med.* **127**, e3-4 (2014).
6. Sanchis-Gomar, F. *et al.* Effects of allopurinol on exercise-induced muscle damage: new therapeutic approaches? *Cell Stress Chaperones* **20**, 3-13 (2015).

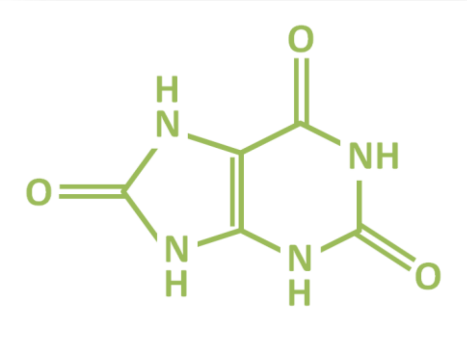

Notes from an Athlete

V.V Mei or Mei Yamaguchi

Mixed Martial Arts Fighter

DEEP JEWELS Atom-Weight Champion, ONE Championship

First of all, I'm very grateful to the Guideline Development Team for giving me this chance to add my comments to the Guideline.

I am a professional mixed martial arts fighter. A few years ago, the results of a blood test showed that I had an abnormally high uric acid level. I was diagnosed with hereditary renal hypouricemia (RHUC). Learning about the characteristics of my disease in more detail in the Guideline helped me to understand the cause of this health problem, from which I have suffered for a long time, and also to know how to cope with it. This information helped me a lot, both daily and in my athletic career.

RHUC patients rarely notice that they have this condition, because they experience no problems in their daily life. However, athletes or people who engage in intense physical activity can face serious health problems if they don't know about this condition. My own experience is that throughout elementary, junior high, and high school, I often felt nauseous after short-distance running and sports tests. My teachers were concerned, and thought it might be due to sudden and vigorous exercise without warm-up, but the actual cause was the heavy load on my kidneys due to RHUC. As an adult, to prepare for mixed martial arts matches, I had to lose weight, which placed great demands on my internal organs. I then engaged in fierce fights. Afterwards, I suffered exercise-induced acute kidney injury (EIAKI), which caused me to feel ill and vomit repeatedly. Sometimes couldn't eat normally for a couple of weeks.

Looking back, I can see that my body was repeatedly sending me warning messages, but not knowing that I had RHUC, I couldn't respond appropriately, and as a result I injured my health many times. After martial arts fights I felt sick, and whenever I saw other fighters staying energetic, even after fights, I often thought that I was getting more tired and injured because I hadn't prepared hard enough for the fights, and blamed myself for my lack of dedication. As the Guideline shows, knowing about RHUC and its effects helped me to make the right treatment decisions, and as a result, I don't feel so sick now during intensive training for fights or even after fights.

I hope that more medical institutions will make use of the information in the Guideline to anticipate and prevent urinary stones and EIAKI, for example, and that they will chose and prescribe painkillers and cold remedies that apply less stress on the kidneys. In addition, I hope that, having read this Guideline, sports medicine practitioners will be better able to

inform patients on what type and intensity of exercise is appropriate for EIAKI patients, provide them with appropriate treatment, and that they will no longer misdiagnose the backache characteristic of EIAKI as simple muscle pain or tiredness.

Last but certainly not least, I'd like to send a message to my fellow sufferers of RHUC. If you focus on preserving your health, you can enjoy life like everyone else, for example, by engaging in ordinary daily activities of course, but also sports, marathons, and even martial arts! It is very important to be aware of your daily levels of physical activity and to remember the characteristics of RHUC. The Guideline tells us, for example, what type of care we need to take before exercise, what intensity of exercise we can stand, and what we need to be aware of after intense physical activity. It is easy to forget, at normal levels of physical activity, that we have RHUC, because it has no symptoms, but to prevent disease complications, this is something we need pay close attention to every day.

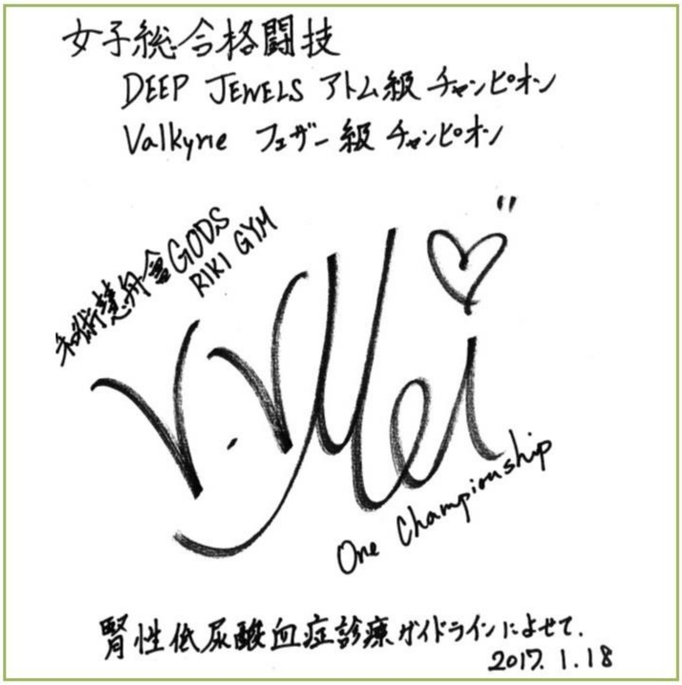

Supplement: Supplementary file 1 — Supplementary material 1 (PDF 6184 KB) [file 13577_2019_239_MOESM1_ESM.pdf]
